# Novel histotypes of sporadic Creutzfeldt–Jakob disease linked to 129MV genotype

**DOI:** 10.1186/s40478-023-01631-9

**Published:** 2023-08-31

**Authors:** Laura Cracco, Gianfranco Puoti, Antonio Cornacchia, Katie Glisic, Seong‑Ki Lee, Zerui Wang, Mark L. Cohen, Brian S. Appleby, Ignazio Cali

**Affiliations:** 1 Department of Pathology and Laboratory Medicine, School of Medicine, Indiana University, Indianapolis, IN 46202 USA; 2 Division of Neurology, University of Campania “Luigi Vanvitelli”, Caserta, Italy; 3 Department of Pathology, School of Medicine, Case Western Reserve University, Cleveland, OH 44106 USA; 4 National Prion Disease Pathology Surveillance Center, Cleveland, OH 44106 USA; 5 Department of Physiology and Biophysics, School of Medicine, Case Western Reserve University, Cleveland, OH 44106 USA; 6 Department of Neurology, School of Medicine, Case Western Reserve University, Cleveland, OH 44106 USA; 7 Department of Psychiatry, School of Medicine, Case Western Reserve University, Cleveland, OH 44106 USA

**Keywords:** Prion protein, Prion, sCJD, Histotype, Codon 129, RT-QuIC

## Abstract

The MV1 and MV2 subtypes of sporadic Creutzfeldt–Jakob disease (sCJD) are linked to the heterozygous methionine (M)/valine (V) polymorphism at codon 129 of the prion protein (PrP) gene. MV2 is phenotypically heterogeneous, whereas MV1, due to its low prevalence, is one of the least well characterized subtypes. In this study, we investigated the biochemical properties of PrP^Sc^ and phenotypic expression of cases diagnosed as sCJD MV1 and MV2. We describe four MV2 histotypes: 2C, with cortical (C) coarse pathology; 2K, with kuru (K) plaque deposits; 2C-K, with co-existing C and K histotypic features; and the novel histotype 2C-PL that mimics 2C in the cerebral cortex and cerebellum, but exhibits plaque-like (PL) PrP deposits in subcortical regions (e.g., basal nuclei, thalamus and midbrain). Histotype prevalence is highest for 2C-K (55%), intermediate for 2C (31%), and lowest for 2C-PL and 2K (7%). Nearly every MV2 case expressed both PrP^Sc^ types, with T2 being the predominant type (“MV2-1”). MV1 cases typically show a rapid disease course (≤ 4 months), and feature the 1C histotype, phenotypically identical to sCJDMM1. Co-existing PrP^Sc^ types, with T1 significantly exceeding T2 (“MV1-2”), are detected in patients diagnosed as MV1 with longer disease courses. We observed four histotypes among MV1-2 cases, including two novel histotypes: 1V, reminiscent of sCJDVV1; 1C-2C, resembling sCJDMM1-2 with predominant MM1 histotypic component; and novel histotypes 1C-2PL and 1C-2K, overall mimicking 1C in the cerebral cortex, but harboring T2 and plaque-like PrP deposits in subcortical regions (1C-2PL), and T2 and kuru plaques in the cerebellum (1C-2K). Lesion profiles of 1C, 1V, and 1C-2C are similar, but differ from 1C-2PL and 1C-2K, as the latter two groups show prominent hippocampal and nigral degeneration. We believe that the novel “C-PL” histotypes are distinct entities rather than intermediate forms between “C” and “C-K” groups, and that 1C-2PL and 1C-2K histotypes may be characterized by different T1 variants of the same size.

**Supplementary Information:**

The online version contains supplementary material available at 10.1186/s40478-023-01631-9.

## Introduction

Sporadic Creutzfeldt–Jakob disease (sCJD) is classified into five major subtypes (MM/MV1, VV1, MM2, MV2, and VV2) based on the pairing of the pathogenic scrapie prion protein (PrP^Sc^) type (1 or 2), and the methionine (M)/valine (V) genotype at codon 129 of the PrP gene (i.e., 129MM, 129MV and 129VV) [34]. In addition to these five sCJD “pure” subtypes, “mixed” subtypes accumulate PrP^Sc^ type 1 (T1) and type 2 (T2) in different ratios, and are classified into MM1-2, MV1-2, VV1-2 [7, 14]. The relative proportion of the co-existing PrP^Sc^ types strongly influences the disease phenotype [7]. In sCJD, proteinase K (PK) cleaves PrP^Sc^ in its “variable region”, which encompasses residues 74 to 102. This proteolytic digestion generates truncated, partially PK-resistant PrP^Sc^ (resPrP^Sc^) fragments of different size [36]. Truncated fragments of ~ 21 or ~ 19 kDa are formed when PK cleaves PrP^Sc^ at glycine 82 or serine 97. These two fragments are unglycosylated isoforms of resPrP^Sc^ T1 (T1^21^) and T2, respectively, and are used as surrogate markers for typing purposes [34–36]. However, the variable region of PrP^Sc^ also provides secondary cleavage sites [32, 36]. Notably, proteolytic digestion of PrP^Sc^ T1 is strongly influenced by the buffer pH [4, 31]. Thus, depending on whether the buffer pH is tittered at 6.9 or 8.0, PK will generate an unglycosylated resPrP^Sc^ fragment of ~ 21 or ~ 20 kDa (T1^20^). Unlike T1, T2 always migrates to ~ 19 kDa [4, 31]. T1^20^ is invariably associated with MM1, MV1, MM1-2 subtypes [7, 8, 31] as well as sCJD-129MM with PrP plaque deposits in the grey matter (p^GM^-CJD) [4]. Furthermore, co-existing T1^21^ and T1^20^ variants, which have strain properties [11], are common in VV1 [14, 31], VV1-2 [14], and in sCJD cases with PrP plaques affecting the white matter (p^WM^-CJD) [4, 5, 24, 27, 41]. Genotype at codon 129 has an effect on both prion disease susceptibility and clinico-pathological phenotype [35, 38]. Homozygous 129MM and 129VV genotypes are more susceptible to prion disease than 129MV heterozygous [16, 33]. In subjects with variant CJD, an acquired prion disease caused by the consumption of bovine spongiform encephalopathy-contaminated beef, the prevalence of 129MM is close to 100% [17]. In genetic prion diseases, the D178N mutation coupled with *cis* 129M is associated with fatal familial insomnia [25] whereas the D178N mutation coupled with *cis* 129V is associated with genetic CJD [22].

Recently, a novel mechanism of phenotypic determination has been described in sCJDMV2 [30]. According to this model, the amount of PrP^C^-129M/-129V that is converted into PrP^Sc^ has a strong effect on the resulting phenotype, which encompasses three histotypes: i) MV2C, which mimics MM2 sCJD, and is characterized by cortical (“C”) spongiform degeneration (SD) with large vacuoles and perivacuolar/coarse PrP deposition; ii) MV2K, identifiable by the presence of kuru (K) plaques and lack of cortical MM2 pathology; and iii) MV2C-K, which includes a spectrum of 2K and 2C mixed histopathological features [1, 30]. These three histotypes are the result of the preferential conversion of PrP^C^-129M into 2C, and of PrP^C^-129V into 2K. Moreover, regional variability in the relative abundance of PrP^C^-129M and -129V results in the distinct severity and topographic distribution of the lesions in 2K and 2C histotypes of 2C-K [19, 30]. Unlike the 2C histotype, which is associated with PrP^Sc^ T2, the western blot profile of 2K shows a ~ 20–19 kDa doublet (T1^20^-T2) with T2 > T1^20^ in the neocortex, and T1^20^ > T2 in the hippocampus and subcortical regions [1, 7, 40]. However, whether (i) a prominent T1^21^ is consistently detected in 2C-K cases, and (ii) intermediate histotypes exist between 2C and 2C-K has not been explored extensively, likely due to 2C and 2C-K with a predominant C component being rare histotypes [1]. Because of its rare occurrence, little is also known about the MV1 subtype. A recent report has contributed to further dissect the histopathology of this subtype, and a small subset of five “sCJDVM1” cases was shown to have clinical, histopathological and molecular features resembling the VV1 subtype [23]. All VM1 cases had ≥ 10 months disease duration, which is significantly longer than that of classical MV1 [21, 35]. Given the high phenotypic heterogeneity of sCJDMV2, and the recent report on VM1, we wondered whether further unrecognized histotypes may exist. To answer this question, we retrospectively examined 110 sCJD cases linked to codon 129MV genotype (sCJDMV) to assess the disease phenotype and molecular features of PrP^Sc^. The large sCJDMV population included 79 MV2 and 31 MV1 cases that were referred to the National Prion Disease Pathology Surveillance Center (NPDPSC) in Cleveland, USA. Sporadic CJDMV2 was classified into 2K, 2C, or 2C-K according to the histopathological features mentioned above. In addition to these well-known histotypes, we identified a novel histotype that resembles 2C in the cerebral cortex and cerebellum, but forms plaque-like PrP deposits in subcortical regions, and more severe lesions in the midbrain. We named this histotype “2C-plaque-like” or “2C-PL”. In cases diagnosed as MV1, the classical histotype with fine SD and diffuse PrP (identified as 1C), is the most common and is typically observed in subjects with short disease duration (≤ 4 months). However, a small proportion of “MV1” cases with slower disease progression harbored small amounts of focally distributed resPrP^Sc^ T2. We identified two novel histotypes in this case cohort. The first one, analogous to 2C-PL, mimics the 1C histotype except for the presence of subcortical plaque-like PrP deposits (1C-2PL histotype). The second, and perhaps the most interesting, is reminiscent of 1C in the cerebral cortex, and of 2K in the cerebellum (1C-2K histotype). Overall, the lesion profiles of 1C-2K and 1C-2PL were similar, and they both differed from those of the other histotypes in the degree of subcortical pathology. Finally, a single case with 31-month disease duration resembled the recently reported VM1 (1V histotype) [23]. Our study defines the histotype prevalence, resPrP^Sc^ type distribution, and clinical features in a large cohort of sCJDMV cases, allowing identification and classification of these novel histotypes by neuropathologists.

## Materials and methods

### Case series

All 110 sCJDMV cases were referred to the NPDPSC between 2001 and 2018. Based on routine western blot analyses of three brain regions, frontal and occipital cortices (FC and OC), and cerebellum (CE), 31 cases were diagnosed as MV1 and 79 as MV2. In this study, we performed a detailed western blot characterization on 27 MV1 and 32 MV2 cases. In MV2 cases (32–35, 55–57, 60–73, 100–110; Additional file 1: Table S1), nine brain regions were examined, including the FC (middle gyrus), temporal cortex (TC), parietal cortex (PC) and visual cortex (VC), hippocampus (HI), striatum (ST), anterior thalamus (TH), the substantia nigra (SN) of midbrain, and CE. The FC (superior gyrus) and non-visual cortex of the occipital lobe (OC) were sampled in two cases, and the entorhinal cortex (EC) in four cases, respectively. In 22 of the 27 MV1 cases undergoing extensive western blot analysis (1–16, 18, 22, 25, 29–31; Additional file 1: Table S1), in addition to the nine brain regions indicated above, we sampled the superior gyrus of FC, the OC and EC, for a total of twelve brain regions/case. In case 22, we sampled the cerebellar hemispheres from four different locations. In other MV1 cases (23, 24, 26–28; Additional file 1: Table S1) we harvested the following brain regions: FC (middle gyrus), OC (23, 24, 26–28), TC (23, 24, 27), ST (23, 28), caudate nucleus (26), TH (23, 24, 27, 28), SN (28), CE n = 3 samples (26–28), CE n = 2 samples (23, 24). The following brain regions were not available: FC (cases 34, 110), TC (cases 9, 104), PC (103, 104), HI (9, 18, 60, 73, 105, 110), ST (9, 72, 104), EC, VC, and OC (case 9), TH (35, 57, 73, 105), SN (9, 18, 34, 56, 60, 100, 102, 104, 105), and CE (1, 34, 56). resPrP^Sc^ was not detected in the OC (60, 64), HI (57), and CE (24, 32, 33, 57, 67–69). Thus, we assessed resPrP^Sc^ typing in a total of 547 samples. Histopathological examination was carried out in all cases, whereas clinical data were reviewed in 80 cases (Additional file 1: Table S1). Six cases (105–110, Additional file 1: Table S1) were not included in the blind estimation of the histotype prevalence because we were aware of the histotype. Moreover, cases diagnosed as MV1-2 by the NPDPSC were not included in this study, as they will be studied separately. Brain tissue from sCJD MM1 and MM1-2 subtypes were used as controls on western blot analysis [7].

### Brain homogenate preparation, PK digestion of PrP^Sc^

Frozen brain tissue was homogenized with 1X lysis buffer (LB) 100 (1X LB100: 100 mM NaCl, 10 mM EDTA, 0.5% NP-40, 0.5% sodium deoxycholate, 100 mM Tris–HCl, pH 8.0) to generate 10% (wt/vol) brain homogenate (BH). In 30 cases (11 MV1, 9 MV2, 3 MM1, 7 MM1-2), brain tissue was homogenized in 1X LB100 tittered at pH 6.9 [31]. Brain homogenates were spun at 1000 × *g* for 5 min (4 °C) and supernatants (S1) were collected. All samples (S1) generated with LB100 pH 8.0 were digested with 10 Units/ml (U/ml) PK (1 h at 37 °C) [PK specific activity was 48 U/mg at 37 °C, with 1 U/ml equal to 20.8 μg/ml PK]. Samples homogenized with 1X LB100 pH 6.9 were digested with 32 U/ml PK for 1 h (37 °C). We also used 5 U/ml PK in one MM1-2 case. Proteolytic digestion was stopped by the addition of 3 mM PMSF, and S1 were then mixed with an equal volume of 2X Laemmli buffer and denatured (100 °C for 10 min).

### Western blot analysis for PrP^Sc^ typing

Denatured proteins were loaded onto 15% Tris–HCl SDS–polyacrylamide gels (W × L: 20 cm × 20 cm; Bio-Rad PROTEAN^®^ II xi cell system), and in 15% Tris–HCl precast gels (W × L: 13.3 cm × 8.7 cm; Bio-Rad Criterion™). Proteins were then transferred onto Immobilon PVDF membranes, blocked with blocking buffer (5% non-fat milk in Tris-buffered saline with Tween-20) and probed with antibodies 3F4 (1:10000), 1E4 (1 µg/ml) or tohoku-2 (1:10000) [4, 7]. Membranes were washed with 1X TBS-T (1X TBS with Tween 20) and probed with a horseradish peroxidase conjugated goat anti-mouse or anti-rabbit antibody (1:3000). PrP proteins were visualized on Kodak films by the ECL Plus as described by the manufacturer [8, 9, 12]. Western blot analysis of cases 23, 24, 26–28 (Additional file 1: Table S1) was carried out using Odyssey near-infrared imaging system (LICOR Biosciences) [4, 10, 13].

### Real-time quaking-induced conversion (RT-QuIC)

Before performing RT-QuIC, we measured the amount of insoluble PrP^Sc^ (obtained at 100,000 × *g*) that was extracted from the thalamus of 1C-2PL and 1C-2K cases. Samples with larger PrP^Sc^ amounts were diluted so that all cases had the same PrP^Sc^ amount, which was determined by western blot analysis. RT-QuIC was carried out as previously described with some modifications [4]. Bacterially expressed, truncated (amino acids 90–231) Syrian hamster recombinant PrP (recHaPrP) was used as substrate; kinetic reactions were average of three reaction wells of sample with 10^–4^, 10^–6^, or 10^–8^ dilution.

### Histology, lesion profile and immunohistochemistry

Formalin-fixed paraffin-embedded tissue sections were deparaffinized, rehydrated, and microwaved with 1.5 mM HCl for antigen retrieval. Sections were immersed in 1X TBS-T. Following incubation with 2.4% hydrogen peroxide (H_2_O_2_) solution, sections were immersed in 1X TBS-T, incubated with 10% normal goat serum for 30 min, and probed with 3F4 antibody (1:1000) for one hour. Sections were washed with 1X TBS-T, incubated with the Dako Envision + System HRP Labelled Anti-Mouse for 30 min, washed with TBS-T, and incubated with Envision Flex DAB (Agilent) for PrP visualization. Ten brain regions were stained with hematoxylin–eosin (H&E) and 3F4 antibody: FC, OC, PC, TC, EC, HI, ST, TH, MB, and CE. Lesion profiles were generated following semi-quantitative assessment of the severity of spongiform degeneration (SD) and gliosis. Gliosis and SD were scored on a 0–3 and 0–4 scale, respectively (0, absent; 1: mild; 2: moderate; 3: severe; 4: status spongiosus for SD). Lesion profiles were carried on 8 brain regions: FC, OC, HI (CA1-CA4), ST, TH, SN, and CE.

### Molecular genetics

DNA was extracted from frozen brain tissue. Genetic analysis was performed to identify possible mutations in the PrP gene (*PRNP*), and to determine the polymorphism at codon 129 of *PRNP*. Genetic analysis was performed as previously described [34, 36].

### Clinical evaluations

Medical records are requested when cases are submitted to the NPDPSC for neuropathological examination, the legal next of kin completes an autopsy consent form that includes information on recognized and possible acquired prion disease risk factors. Data was collected on demographics such as gender, race/ethnicity, age at disease onset, and disease duration. Medical records, including past medical and surgical histories, as well as other risk factors for the development of an iatrogenic prion disease, clinical symptoms, family history, and diagnostic test results were obtained and reviewed by a clinician, but were not available in all cases.

### Image acquisition and statistical tests

All microphotographs were taken with Leica DFC 425 digital camera that is mounted on a Leica DM 2000 microscope. When needed, densitometric analysis of resPrP^Sc^ unglycosylated fragments was carried out with LI-COR application software 3.0. Statistical significance was determined by (i) Student’s *t*-test (two-tailed) for age at onset, disease duration, and total tau; (ii) Chi-square test for gender, race, MRI and positive CSF 14–3-3; (iii) Fisher’s exact test for EEG with PSWCs (Table 1); (iv) One-way ANOVA for lesion profiles (Additional file 1: Table S3) and RT-QuIC. Statistical analyses as well as charts were generated using GraphPad Prism 9.5.0.

**Table 1:** Clinical features of sCJDMV histotypes

Histotype	N cases	Age at onset (years)	Disease duration (months)	Female	White	EEG with PSWC_s_	MRI suggestive of CJD	Positive CSF 14–3-3	Positive CSF RT-QuIC	Total tau [pg/ml] × 1000
1C	21	69 ± 11^a^	7 ± 7^a^	62^b^ 13/21 ^c^	80^b^ 16/20 ^c^	71^b^ 12/17 ^c^	68^b^ 13/19 ^c^	92^b^ 11/12 ^c^	100^b^ 1/1 ^c^	9.7 ± 6.3^a^ (n = 10)
1V	1	64	31	100 1/1	0 0/1	0 0/1	100 1/1	100 1/1	na	2.4 (n = 1)
1C-2PL	3	74 ± 5	12 ± 7	0 0/3	100 2/2	50 1/2	33 1/3	100 1/1	na	13 ± 14 (n = 2)
1C-2K	4	71 ± 4	12 ± 2	0 0/4	100 3/3	25 1/4	50 2/4	100 1/1	na	7.3 (n = 1)
1C-2C	2	71 ± 1	7 ± 4	0 0/2	100 2/2	50 1/2	50 1/2	100 1/1	na	5.3 (n = 1)
All (MV1/MV1-2)	31	70 ± 9	9 ± 8	45 14/31	82 23/28	58 15/26	62 18/29	94 15/16	100 1/1	9.2 ± 6.8
2C	23	70 ± 9	27 ± 16	56 13/23	90 19/21	9 1/11	90 18/20	33 3/9	100 10/10	1.5 ± 0.8 (n = 14)
2C-PL	5	72 ± 7	14 ± 6	80 4/5	100 5/5	0 0/1	100 2/2	100 1/1	na	1.9 ± 1.4 (n = 2)
2C-K	14	69 ± 8	14 ± 8	36 5/14	100 14/14	11 1/9	36 4/11	50 1/2	na	2.4 ± 1 (n = 2)
2K	11	67 ± 8	14 ± 7	27 3/11	100 11/11	0 0/10	90 9/10	86 6/7	na	na
All (MV2-1)	53	69 ± 8	20 ± 13	47 25/53	96 49/51	6 2/31	77 33/43	58 11/19	100 10/10	1.6 ± 0.9
*P* values^d^		NS^e^	< 0.0001^e^	NS^f^	NS^f^	< 0.0002^g^	NS^f^	< 0.02^f^		< 0.0008^e^

*EEG* Electroencephalogram; *PSWC* Periodic short-wave complexes; *MRI* Magnetic resonance imaging; *CSF* Cerebrospinal fluid

MRI: 2C vs. 2C-K, *P* < 0.004 (Fisher’s exact test)

^a^Expressed as mean ± SD and ^b^percentage. ^c^Cases with the feature listed/total cases examined; ^d^It compares MV1/MV1-2 and MV2-1 cases; ^e^Student’s *t*-test; ^f^Chi-square test; ^g^Fisher’s exact test

## Results

**Fig. 1:**
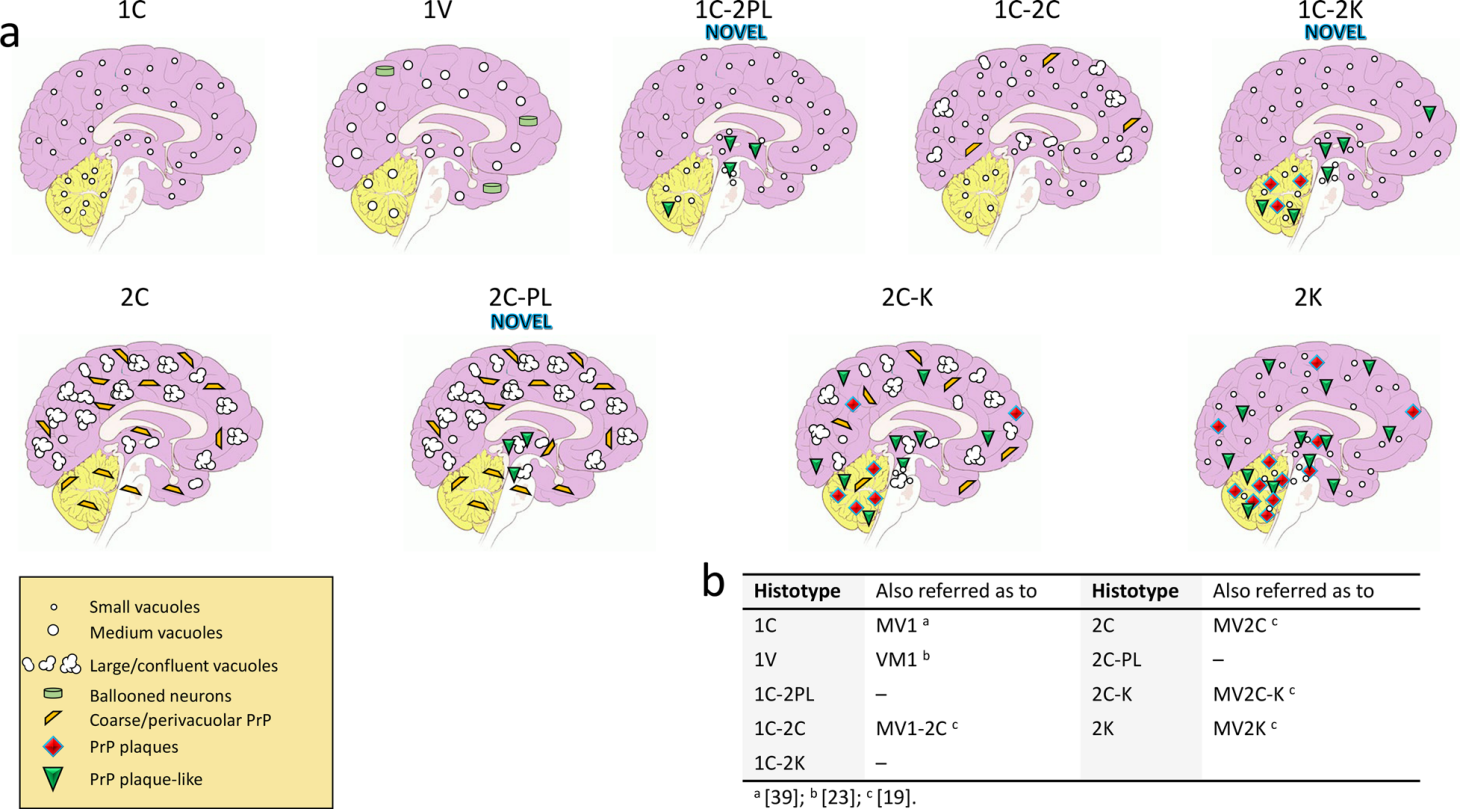
Schematic representation of the histotypes identified in this study. **a** The first raw identifies histotypes associated with MV1 and MV1-2; the second row refers to MV2-1. **b** Nomenclature used in this and other studies

We identified 3 novel histotypes in the retrospective histopathological evaluation of 110 sCJDMV cases (Fig. 1). Twenty-seven of the 31 MV1 cases were re-characterized by (i) high resolution gel electrophoretic analysis of various brain regions, and (ii) standard and type-selective PrP antibodies. Sixteen of these 27 cases (60%) are “pure” T1 (no T2 detected), have disease duration ranging from 1 to 4 months, and only the 1C histotype present. In the remaining 11 cases (40%), both resPrP^Sc^ types are present, with T1 prominent resPrP^Sc^ type (identified here as “MV1-2”). In these cases, disease duration is typically ≥ 10 months. In this MV1-2 group, the 1C histotype co-exists with the -2PL (1C-2PL), -2K (1C-2K), or -2C (1C-2C) histotype (Fig. 1). Moreover, the 1V histotype belongs to MV1-2 [23].

In the 32 MV2 cases with extensive western blot characterization, both resPrP^Sc^ types co-exist in 31 cases (97%). Because T2 is the predominant resPrP^Sc^ type, we refer to this group as “MV2-1”. For convenience, the nomenclature “MV2-1” is used also for the only case with 2C histotype and “pure” T2 on western blot. Thus, 2C, 2C-K and 2K, as well as the novel 2C-PL histotype, are included in MV2-1 (Fig. 1).

### Histopathology of sCJD MV1 and MV1-2

#### 1C histotype

**Fig. 2:**
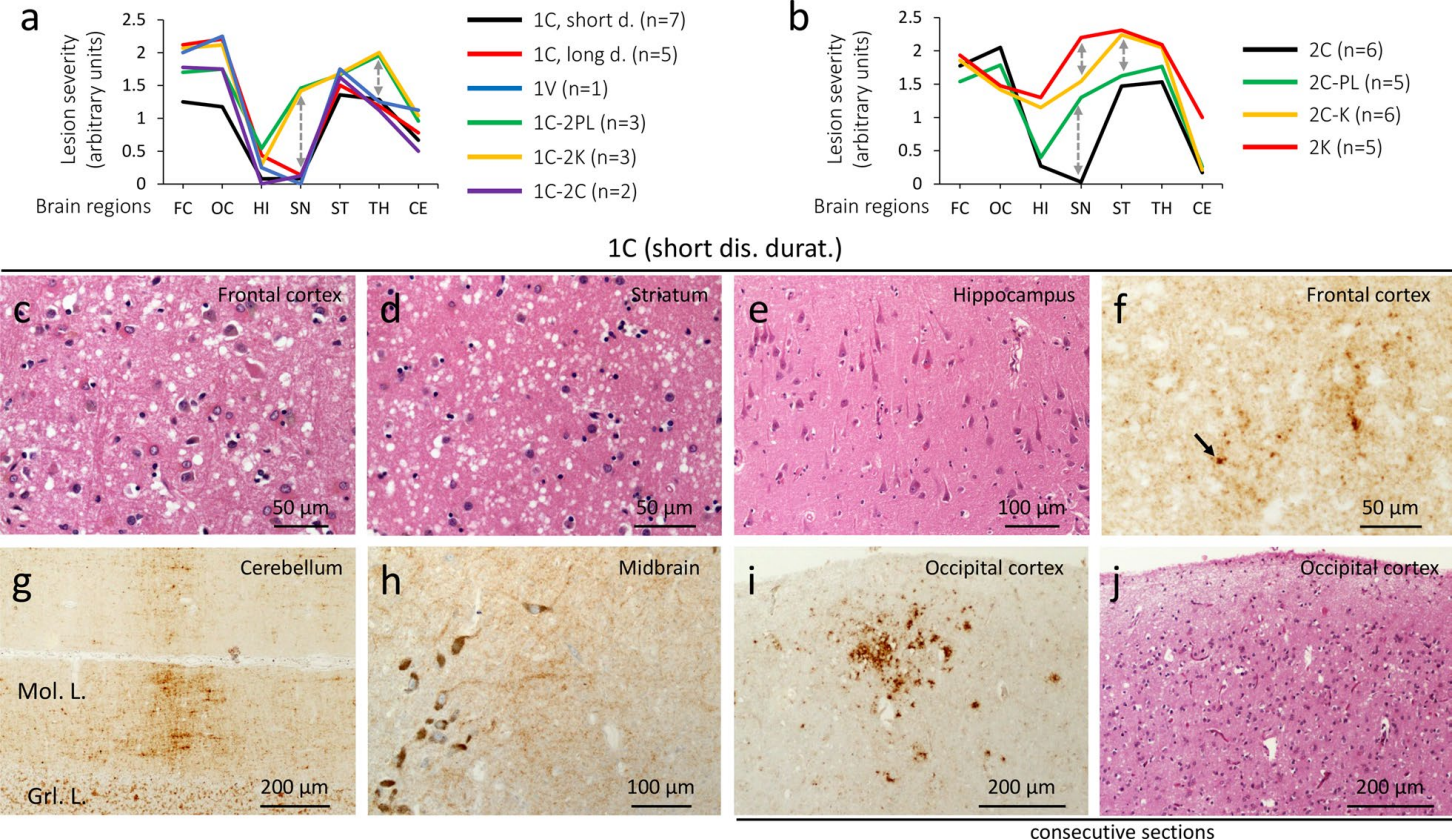
Lesion profiles of 9 sCJDMV histotypes, and histopathology of 1C. **a** sCJD MV1/MV1-2 can be divided into two major groups. The first one includes 1C, 1V and 1C-2C histotypes, characterized by the sparing of the substantia nigra (SN) and less severe pathological changes in thalamic (TH) nuclei; the second group encompasses 1C-2PL & 1C-2K showing more severe lesions of SN and TH, and accumulation of plaque-like PrP deposits (1C-2PL) and PrP plaques (1C-2K). **b** Lesion profiles of MV2-1 show three major groups: 2C with sparing of SN, 2C-PL with mild lesions of SN, and 2C-K & 2K with overall different lesion profiles, and marked hippocampal pathology; the 2C-K histotype shows C and K histotypic features in the proportion of ~ 50% each. Dashed double arrows point to differences in severity in key brain regions. **c**–**e**, **j** Hematoxylin–eosin (H&E). **f**–**i** PrP immunostaining. **c**–**e** Spongiform degeneration affecting the neocortex (**c**) and subcortical regions (**d**) regions, but not the hippocampus (**e**). **f**–**h** Diffuse PrP; arrow, **f** a larger PrP granule. Mol. L.: Molecular layer; Grl. L.: granular layer. **i** A focus of coarse PrP. **j** H&E preparation stained for PrP in **i**; antibody: 3F4; dis. durat.: disease duration

The lesion profile shows moderate damage (spongiform degeneration, and gliosis) in the cerebral cortex, striatum, anterior thalamus, with mild cerebellar degeneration (Fig. 2a, c, d). The hippocampus and substantia nigra are not affected (Fig. 2a and e). Vacuoles are predominantly of small in size, with occasionally medium-size vacuoles in two cases with long duration (≥ 10 months) (Additional file 1: Table S2). Cases with long survival also show significantly more cerebral cortical degeneration (Fig. 2a). PrP immunostaining (IHC) reveals the diffuse or “synaptic” pattern with scattered clusters of larger PrP granules throughout the brain (Fig. 2f–h; Additional file 1: Table S2) [30]. In three cases, rare foci of coarse PrP (cPrP) are seen in the cerebral cortex (Fig. 2 i and j; Additional file 1: Table S2).

#### 1V histotype

The lesion profile is similar to that of 1C (Fig. 2a); however, the presence of medium-size vacuoles and cortical ballooned neurons are distinctive features of this histotype (Additional file 2: Fig. S1a and b) [23]. PrP staining is very fine in the cerebral cortex and subcortical regions (Additional file 2: Fig. S1c and d); the classic “brush stroke-like” pattern is found in the cerebellum (Additional file 2: Fig. S1e; Additional file 1: Table S2); the midbrain is spared (Additional file 2: Fig. S1f).

#### 1C-2PL histotype

**Fig. 3:**
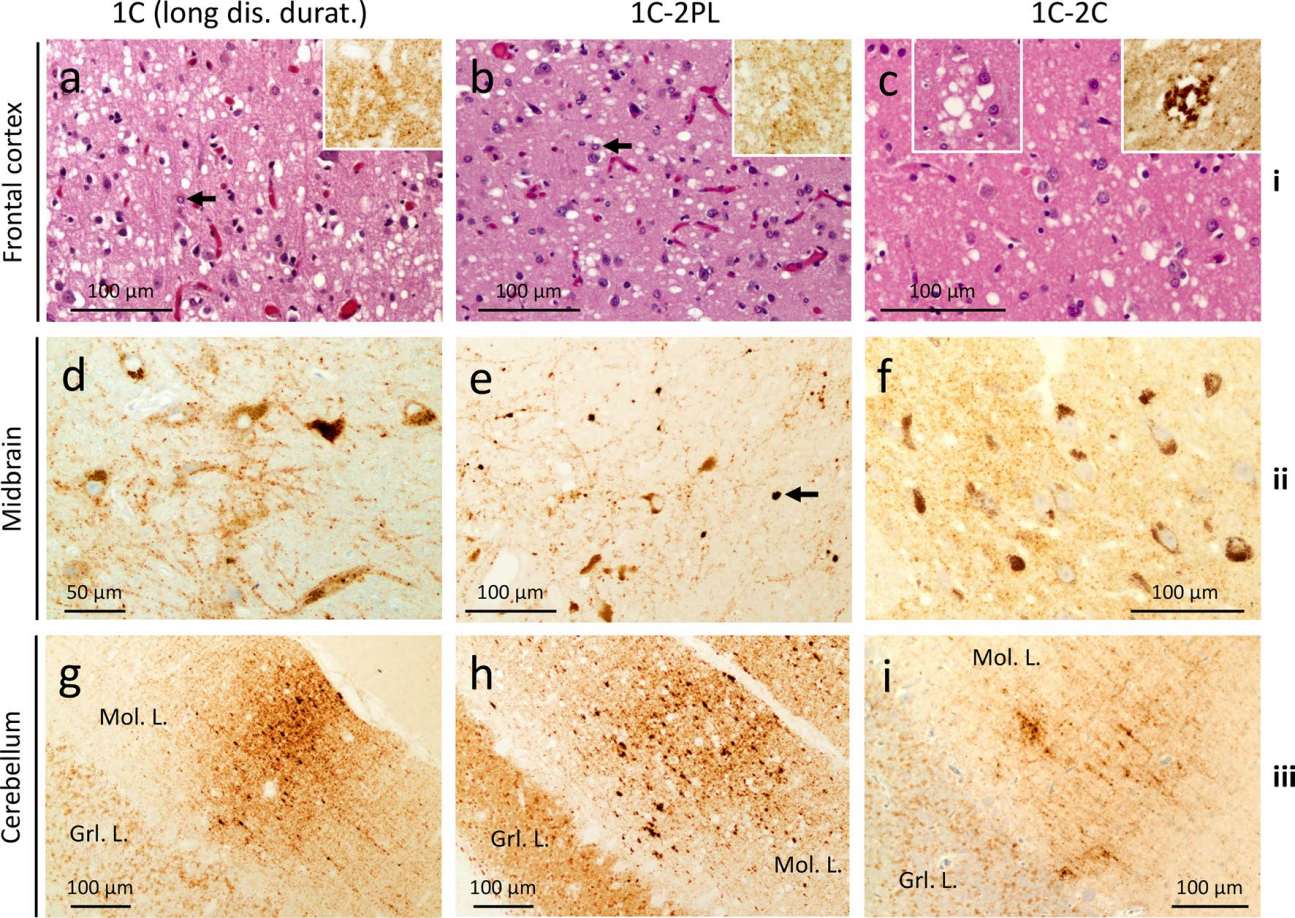
Histopathology of 1C, 1C-2PL and 1C-2C. row **i** H&E. rows **ii**, **iii** and insets in row **i** PrP immunostaining. **a**, **b** Spongiform degeneration (SD) and gliosis; arrows: astrocytes; insets: diffuse PrP. **c** SD with small and occasionally large vacuoles; inset (left): large vacuoles; inset (right): perivacuolar PrP. **d** and **f** Diffuse PrP. **e** Plaque-like PrP; arrow: a plaque-like PrP formation. **g**-**i** “Brush stroke-like” PrP affecting the molecular layer (Mol. L.); Grl. L.: granular layer; antibody: 3F4; dis. durat.: disease duration

Cortical severity is intermediate compared with 1C with short (≤ 4 months) and long disease duration; the substantia nigra and thalamus are more severely affected (Fig. 2a; Additional file 1: Table S3). A further distinctive feature is the presence of plaque-like PrP in subcortical regions, and, in one case, in the cerebellum (Additional file 1: Table S2; Fig. 3e).

#### 1C-2K histotype

**Fig. 4:**
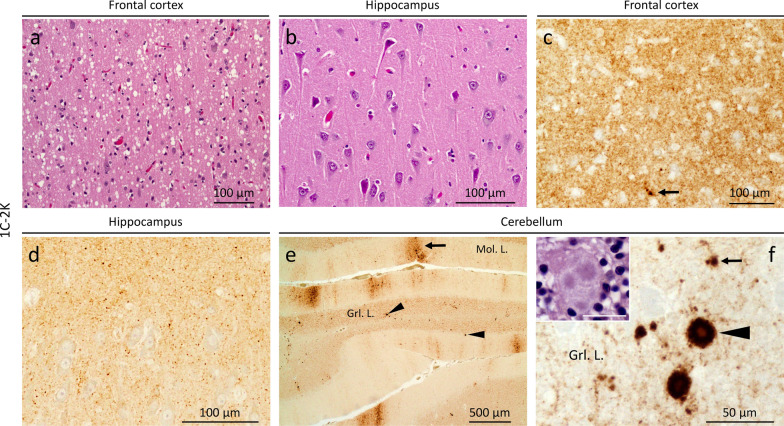
Histopathology of 1C-2K. **a** and** b**, inset in** f** H&E. **c**–**f** PrP immunostaining. **a** Small and mid-size vacuoles. **b** Lack of spongiform degeneration in the CA4 region of the hippocampus. **c** Diffuse PrP immunostaining; arrow: larger PrP granules. **d** Granular PrP staining. **e** “Brush stroke-like” (arrows) and PrP plaques (arrowheads); Mol. L.: molecular layer; Grl. L.: granular layer. **f** PrP plaque (arrowhead) and plaque-like (arrow) deposits; inset: a cluster of the kuru plaques; bar size: 20 µm; antibody: 3F4

Overlaps that of 1C-2PL (Fig. 2a; Additional file 1: Table S3) with scattered medium-size vacuoles in the cerebral cortex (Fig. 4a, Additional file 1: Table S2); the hippocampus is essentially spared (Figs. 2a and 4b). Kuru plaques are found in the cerebellum, and are similar to those of 2K histotype (Fig. 4f; Additional file 1: Table S2). PrP IHC reveals a granule-like PrP throughout the brain (Fig. 4c and d), and both “brush stroke-like” PrP and plaque staining in the cerebellum (Fig. 4e and f). Plaque-like PrP deposits affecting the subcortical regions, and the cerebral cortex in one case (Additional file 1: Table S2). Rare foci of cPrP are noted in 2 cases (Additional file 1: Table S2).

#### 1C-2C histotype

The lesion profile mimics that of 1C (Fig. 2a). This histotype resembles the MM1-2 with MM1 predominant histotypic component (Fig. 3c). The area of the cerebral cortex occupied by cPrP ranges from 5 to 15% (Additional file 1: Table S2). A diffuse PrP pattern is present in the subcortical regions and cerebellum (Fig. 3f and i).

### Histopathology of sCJD MV2-1

#### 2C histotype

**Fig. 5:**
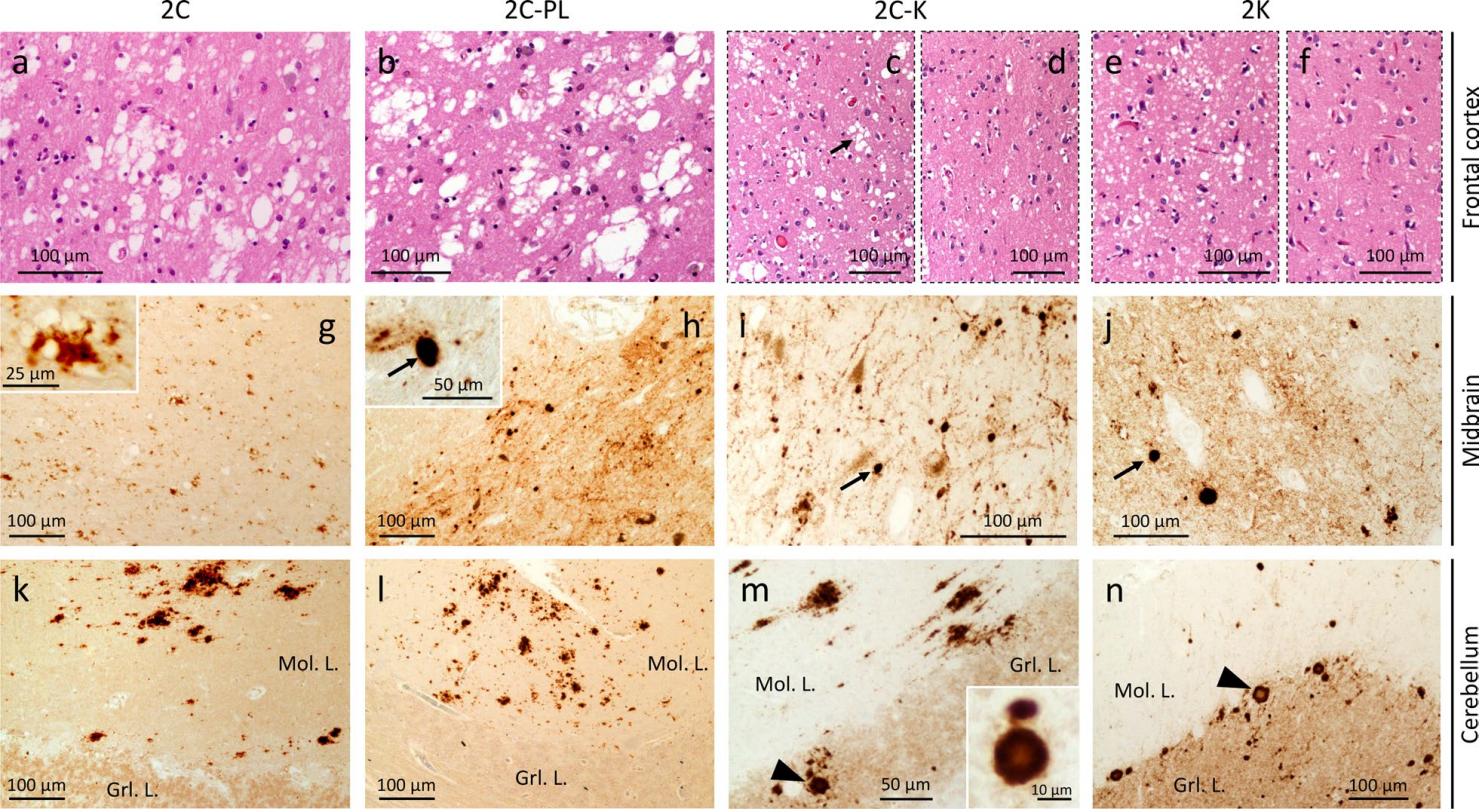
Histopathology of 2C, 2C-PL, 2C-K and 2K. **a**–**f** H&E.** g**–**n** PrP immunostaining. **a**, **b** Large and confluent vacuoles. **c**, **e** Deep layers with small and large vacuoles (**c**), and small vacuoles (**e**); arrow, **c** large vacuoles. **d**, **f** Superficial layers with reduced vacuole density. **g** Coarse PrP; inset, large magnification of perivacuolar PrP. **h**–**j** Plaque-like PrP (arrows) in a background of diffuse PrP and/or PrP arrays; inset, **h** plaque-like PrP (arrow). **k**, **l** Coarse PrP; Mol. L: Molecular layer; Grl L: granular layer. **m**, **n** Coarse PrP deposits (**m**) and PrP plaques (arrowhead) (**m**, **n**); inset, **m** a PrP plaque; antibody: 3F4

Overlaps 1C and 1C-2C (Fig. 2b and Additional file 3: Fig. S2a). Large, confluent vacuoles are present within the neocortex and subcortical regions (Fig. 2b, Fig. 5a; Additional file 1: Table S3). Coarse and perivacuolar PrP patterns are seen throughout the brain (Fig. 5g; Additional file 1: Table S4). Cerebellar cPrP accumulates in ~ 90% of the cases (Fig. 5k; Additional file 1: Table S4).

#### 2C-PL histotype

Resembles that of 1C-2PL (Additional file 3: Fig. S2b). Unlike in the 2C histotype, the substantia nigra is markedly affected (Fig. 2b), and accumulates plaque-like PrP (Fig. 5h; Additional file 4: Fig. S3a; Additional file 1: Table S4). The cerebellum shows cPrP deposits, and plaque-like PrP in one case (Fig. 5l; Additional file 4: Fig. S3b; Additional file 1: Table S4).

#### 2C-K histotype

The lesion profile of this large group (and of 2K, see below), differs significantly from the other histotypes, including: (1) a more severe pathology of the frontal cortex compared to that of the occipital cortex; (2) marked involvement of the hippocampus; and, (3) severe striatal pathological changes (Fig. 2b). The 2C-K histotype also differs from 1C-2K (Additional file 3: Fig. S2c). Spongiosis consists of a mixture of small and large vacuoles in different ratios, with clusters of large vacuoles occupying deeper cortical layers in some cases (Fig. 5c and d). Kuru plaques are easily identifiable in the cerebellar cortex, and occasionally within the cerebral cortex. PrP IHC shows coarse/perivacuolar (2C) and diffuse laminar (or pseudolaminar), perineuronal and plaque-like PrP (2K) patterns [30]. The percentage of cPrP in the cerebral cortex ranges from ~ 1% to 100% (Additional file 1: Table S4). If the 2C-K population is divided in two groups according to cPrP percentage being greater or smaller than 55% (mean cut-off), disease duration is ~ 8 months shorter in subjects with low cPrP (18 vs. 10 months; *P* < 0.02) (Additional file 5: Fig. S4; Additional file 1: Table S4). Cases with less than 55% cPrP also show more severe PrP plaque pathology (data not shown). Furthermore, 2C-K patients with cerebellar cPrP have more cPrP in the cerebral cortex compared to those lacking cerebellar cPrP (75 ± 27% vs. 28 ± 29%, *P* < 0.0001) (Additional file 1: Table S4).

#### 2K histotype

The lesion profile mimics that of 2C-K except for a more severe involvement of the substantia nigra (Fig. 2b; Additional file 1: Table S3). SD is laminar or pseudolaminar in the cerebral cortex; large vacuoles are absent (Fig. 5e and f). Kuru plaques are seen within the cerebellum, subcortical regions, and (to a lesser extent) the cerebral cortex. PrP IHC shows plaque-like, perineuronal, diffuse (laminar and/or pseudolaminar) and plaque patterns (Fig. 5j and n; Additional file 1: Table S4) [19].

### Histotype prevalence and clinical features

**Fig. 6:**
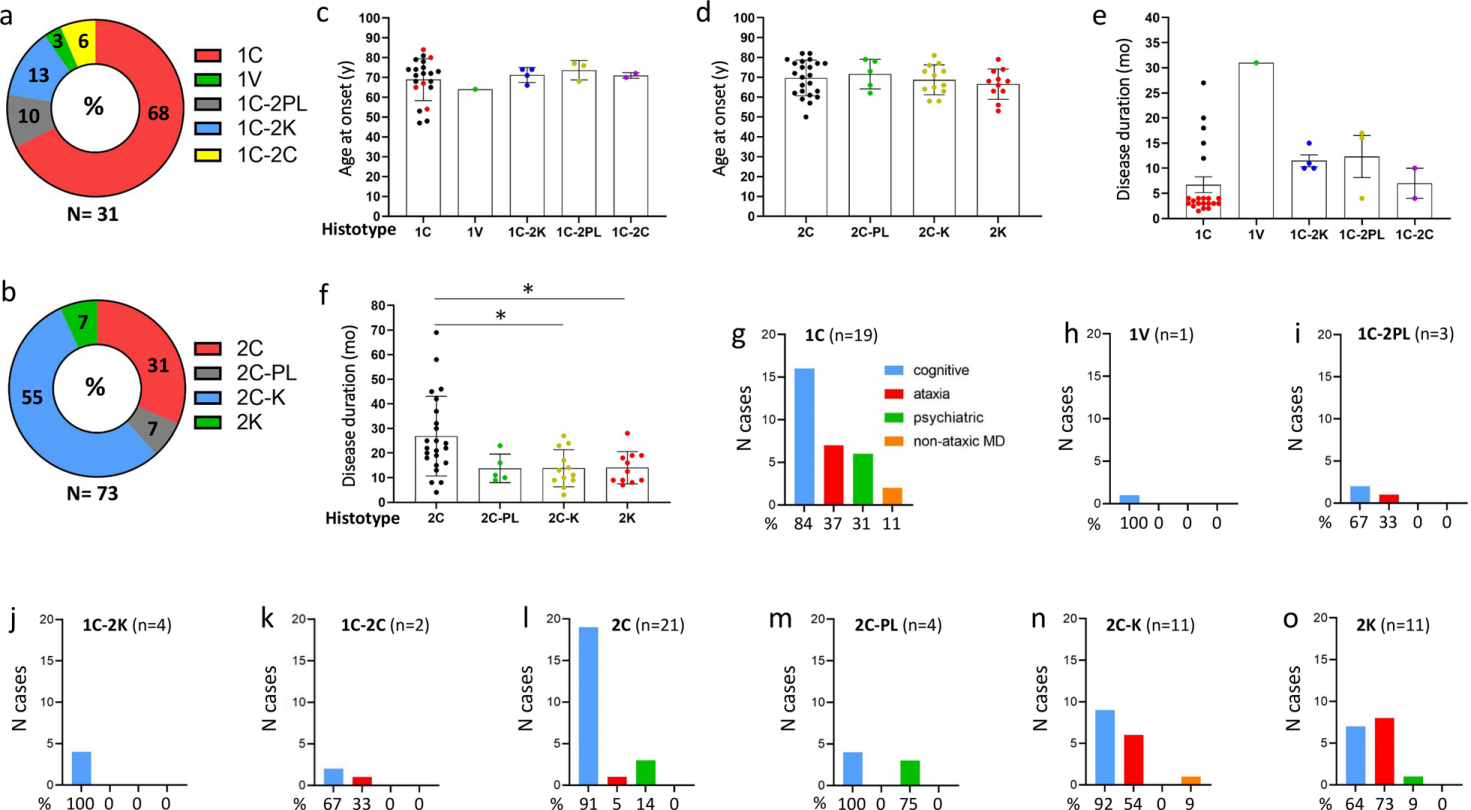
Ring doughnut charts depicting distributions of sCJDMV histotypes and clinical features at presentation.** a** Of the 31 cases from combined MV1/MV1-2 subtypes, 21 (68%) belong to the 1C histotype, and only one (3%) to the 1V histotype. 1C-2PL, 1C-2K and 1C-2C histotypes account for 10%, 13% and 6%, respectively. **b** Of 73 MV2-1, 2C-K (55%) and 2C (31%) are the most common histotypes; with 7% prevalence, 2C-PL and 2K are the rarest histotypes. **c** and **d** The mean age at onset is 70 years in MV1-2 and MV2-1. **e** The 1C histotype includes cases with short (red dots) and long (black dots) disease course. Unlike most 1C cases, disease duration is longer in 1V, 1C-2PL, 1C-2K and 1C-2C histotypes. **f** 2C histotype shows the longest disease duration; *P < 0.03. **g**–**o** With the exception of the 2K histotype (**o**), cognitive decline is the most common symptom at disease onset (**g**–**n**)

In combined MV1 and MV1-2 groups, 1C is the most common histotype (68%); the 1C-2K, 1C-2PL, 1C-2C and 1V histotypes account for 13%, 10%, 6%, and 3%, respectively (Fig. 6a). In MV2-1, 2C-K and 2C (55% and 31%) are more common than 2C-PL and 2K (7% each) (Fig. 6b). Age at onset ranges from 64 years in 1V to 74 ± 5 years (mean ± SD) in 1C-2PL (Fig. 6c and d). In MV1/MV1-2, disease duration is shortest for 1C and 1C-2C (~ 7 months), intermediate in 1C-2K and 1C-2PL (~ 12 months), and longest in 1V (31 months) (Fig. 6e). In MV2-1, the 2C histotype stands out with disease duration twice as long as in any other group (2C vs. combined 2C-PL, 2C-K and 2K: 27 vs. 14 months, *P* < 0.0004) (Fig. 6f). With the exception of the 2K histotype, which has a larger percentage of cases presenting with ataxia (73%), all other sCJDMV histotypes demonstrate a preponderance of cognitive symptoms at clinical presentation (Fig. 6g–o). The sCJDMV histotype with the most variable clinical presentation is 1C, though 84% of these cases still present with cognitive symptoms. Considered as a whole, MV1/MV1-2 and MV2-1 subtypes differ in: (i) mean disease duration shorter in MV1/MV1-2 cases than MV2-1 cases (9 ± 9 vs. 20 ± 13 months; *P* < 0.0001) (Table 1); (ii) EEG, which rarely demonstrate PSWCs in MV2-1 compared to MV1/MV1-2 cases (6% vs. 58%, *P* < 0.0002); (iii) MV1/MV1-2 cases having larger percentage of CSF 14–3-3 positive cases (94% vs. 58%, *P* < 0.02) and higher mean CSF total tau levels (9200 pg/ml vs. 1600 pg/ml, *P* < 0.0008) compared to MV2-1 (Table 1). No differences were noted between the two groups for CSF PrP RT-QuIC and percentage of brain MRIs typical for CJD.

### ***Typing and brain distribution of resPrP***^***Sc***^

#### 1C histotype

**Fig. 7:**
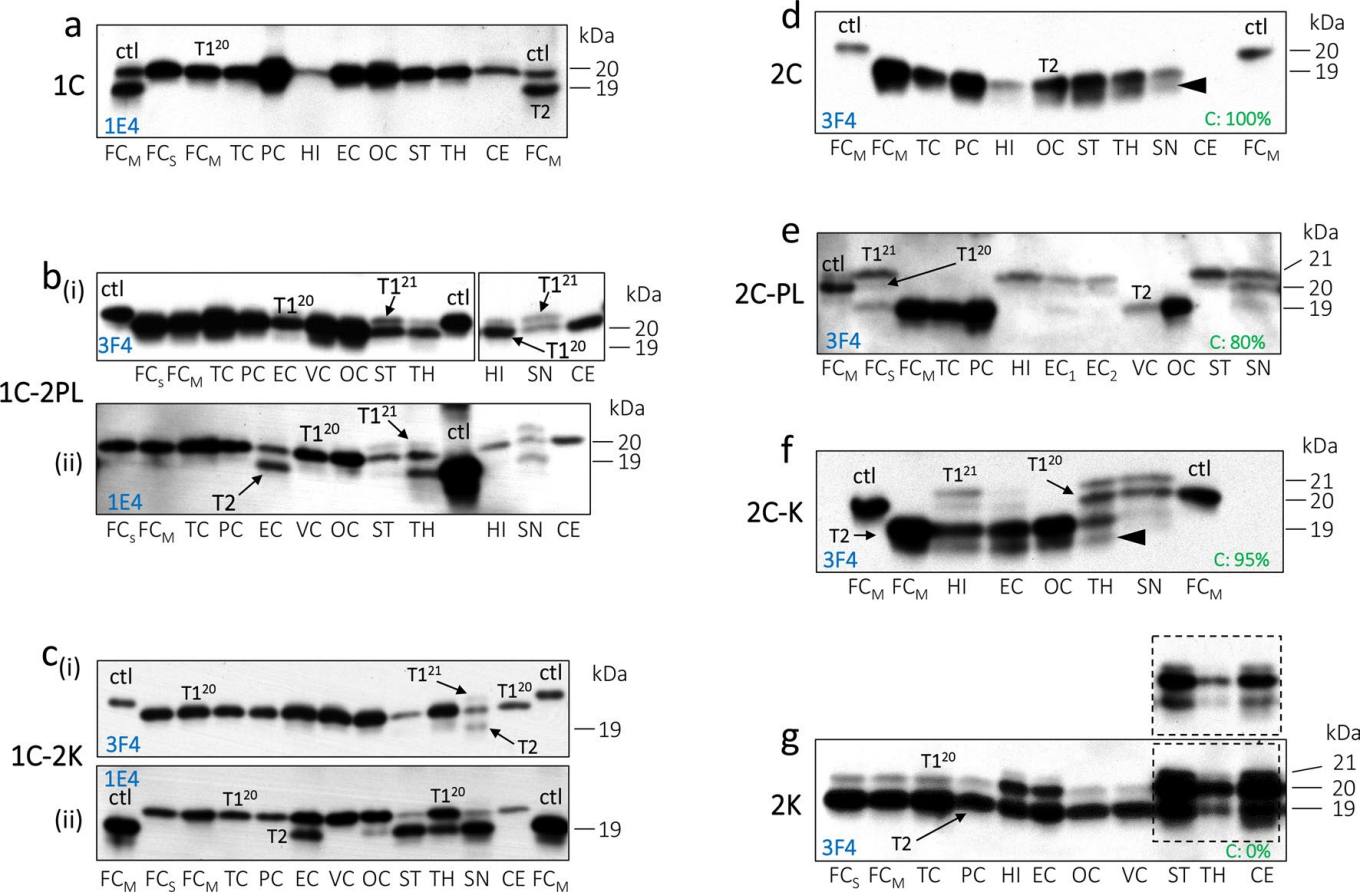
Western blot profiles of sCJDMV resPrP^Sc^. Only the unglycosylated isoform of resPrP^Sc^ is shown for convenience. **a** 1C histotype with short disease duration harbors T1^20^; control (ctl): T1^20^-T2. **b**(**i**) 1C-2PL featuring T1^20^ in the cerebral cortex, and T1^20^ co-existing with a minor T1^21^ fragment in subcortical regions; ctl: T1^21^. **b**(**ii**) T2 is detected in the entorhinal corte (EC), and subcortical regions.** c**(**i**) In 1C-2K T1^20^ is detected in all brain regions, T1^20^-T2 in the SN; Ctl: T1^21^. **c**(**ii**) T2 is detected in the EC, occipital cortex (OC), and subcortical regions; ctl: T2. **d** 2C with “pure” T2 detected in all brain regions; ctl: T1^20^; arrowhead: lower resPrP^Sc^ fragment of ~ 18 kDa. **e** 2C-PL with co-existing T1^21^ and T1^20^ variants, and predominant of T1^21^; ctl: T1^20^. **f** 2C-K with predominant T1^21^ in hippocampus (HI), and predominant T1^20^ in the EC, and subcortical regions; ctl: T1^20^; arrowhead: lower resPrP^Sc^ fragment of ~ 18 kDa. **g** 2K featuring T1^20^-T2 with a prominent T2 in cerebral cortex; inset: shorter time of film exposure. FC_s_: frontal cortex, superior gyrus; FC_M_: FC, middle gyrus; TC: temporal cortex; PC: pariental cortex; VC: visual cortex; ST: striatum; anterior thalamus (TH); substantia nigra (SN) of midbrain; CE: cerebellum. **d**–**g** Percentage of cortical coarse PrP is indicated in green

Cases with short disease duration are associated with resPrP^Sc^ T1^20^ in all brain regions, even when blots are probed with 1E4 and tohoku-2 antibodies, which bind preferentially (1E4) or selectively (tohoku-2) to T2 (Fig. 7a; Additional file 6: Fig. S5; Additional file 1: Table S5) [7]. PK-resistant PrP^Sc^ T1 appears as T1^21−20^ at buffer pH 6.9 or as T1^20^ at buffer pH 8.0 (Additional file 7: Fig. S6a). In individuals with long disease course, western blot analysis on routine examination of 3 brain regions carried out at the NPDPSC (see materials and methods) reveals T1^20^. However, a minor T2 component is detected in one case with 27-month disease duration that was re-assessed by 1E4 in this study (data not shown).

#### 1V histotype

T1^20^ is detected in all brain regions. A minor T2 component is found only after probing with 1E4 (Additional file 8: Fig. S7a and b).

#### 1C-2PL histotype

These cases harbor T1^20^ in the cerebral cortex and cerebellum, and a minor T1^21^ component, co-existing with T1^20^, in subcortical regions (Fig. 7b). T2 is predominantly found in the subcortical regions, and never in the cerebellum (Fig. 7b and Additional file 6: Figs. S5a, Additional file 9: S8a).

#### 1C-2K histotype

T1^20^ is ubiquitous, T1^21^ is underrepresented, and T2 is found predominantly in subcortical regions (Fig. 7c, Additional file 6: Fig. S5, Additional file 7: Fig. S6b, Additional file 9: Fig. S8b). At buffer pH 6.9, the unglycosylated resPrP^Sc^ T1 isoform migrates to ~ 21 kDa in the cerebral cortex, and to ~ 20 kDa in the cerebellum (Additional file 7: Fig. S6b).

#### 1C-2C histotype

T1^20^ co-exists with a minor T2 component that is best represented in the cerebral cortex (Additional file 7: Fig. S6c and Additional file 9: Fig. S8c). As in MM1-2, gel mobility of T1 is influenced by the buffer pH (Additional file 7: Fig. S6c and d).

#### 2C histotype

Detailed western blot analysis of four cases reveals the presence of a minor T1 component in three (75%) (Additional file 1: Table S5). In this group, “pure” T2 (i.e., T1 is undetected) is found in > 90% of the cortical regions, and only in ~ 40% of the subcortical regions (Additional file 1: Table S6). A predominant T1^21^ (T1^21^: T1^21^ > T1^20^) is found in one case (Additional file 1: Table S5). As mentioned above, only one case harbors “pure” T2 in all brain regions (Fig. 7d). T2 is not affected by the buffer pH (Additional file 7: Fig. S6a).

#### 2C-PL histotype

The prevalence of “pure” T2 is high in the cerebral cortex (75%), and low (14%) in subcortical regions; “pure” T2 is never detected in the cerebellum (Additional file 1: Table S6). In two cases, T1^21^ is well represented in the cerebral cortex and subcortical regions (Additional file 1: Table S5; Fig. 7e). Gel mobility of T1 variants and T2 is not affected by the buffer pH (Additional file 7: Fig. S6e).

#### 2C-K histotype

This group is characterized typically by a co-occurrence of resPrP^Sc^ T1^20^ and T2. Prevalence of “pure” T2 is low in the cerebral cortex (27%) and absent in subcortical regions (Fig. 7f; Additional file 1: Table S6). In four cases, T1^21^ is found in the cerebral cortex (29%) and subcortical regions (8%) (Additional file 1: Table S6). Gel mobility of T1 and T2 is not affected by the buffer pH (Additional file 7: Fig. S6f). In the four cases with T1^21^, the 1C histotype is detected only in one (Additional file 1: Table S4). In this patient, diffuse and cPrP co-exist, with cPrP occupying only 15% of the cerebral cortex. In the other three cases, cPrP burden is greater than 80% (Additional file 1: Table S4).

#### 2K histotype

T1^20^-T2 is unequivocally the molecular signature of this group as T1^21^ is absent or under-represented (Fig. 7g; Additional file 1: Table S5). T2 is better represented in the cerebral cortex and cerebellum (~ 80%) (Additional file 1: Table S6). In subcortical regions, T1^20^ (82%) overrun T2 (18%) (Additional file 1: Table S6). T1^20^-T2 is not influenced by the buffer pH (Additional file 7: Fig. S6g) [31].

### RT-QuIC of 1C-2PL and 1C-2K histotypes

RT-QuIC was used to determine the seeding activity of thalamic PrP^Sc^. We used the thalamus because it (i) shows the “plaque-like PrP” pattern, (ii) expresses PrP^Sc^ types 1 and 2 in both groups, and (iii) lacks kuru plaques in the 1C-2K histotype. We normalized all samples to the same amount of PrP^Sc^ (see methods). Kinetic profiles of 1C-2PL and 1C-2K are similar at 10^–4^ and 10^–6^ dilutions (Additional file 10: Fig. S9a and b). However, PrP^Sc^ from 1C-2K seeds recHaPrP at a faster rate, and reaches the plateau earlier at 10^–8^ dilution (Additional file 10: Fig. S9c).

### Overall distribution of T1 variants and 1C histotype in sCJDMV2-1

T1^21^ is found in 7 of 21 (33%) of the MV2-1 cases with a C component (2C-K, 2C-PL and 2C) that underwent extensive western blot examination (Additional file 1: Tables S4 and S5). The 0% T1^21^ prevalence in cases of 2K histotype increases to 29% in cases of 2C-K histotype, and up to ~ 45% in the case-cohort free of kuru plaques (combined 2C and 2C-PL) (Additional file 1: Table S4). Thus, distributions of T1^21^ and that of a prominent T1^20^ (T1^20^ > T1^21^) is opposite, as the prevalence of the latter is 100% in 2K, 71% in 2C-K, and 55% in 2C-PL and 2C combined histotypes (Additional file 1: Table S4). Thus, while T2 is the major resPrP^Sc^ type of MV2-1, T1^20^ is invariably present across all histotypes and is better represented than T1^21^. 8 of the 68 (12%) MV2-1 cases with a 2C component (2C, 2C-PL, and 2C-K) show a focal 1C histotypic feature in the cerebral cortex, and 3 cases in the cerebellum (Additional file 1: Table S4). A trend for statistical significance exists when comparing the percentage of cPrP deposits in the cerebral cortex of MV2-1 cases with (50%) or without (72%) 1C histotype (*P* = 0.061). These data explain why the 1C histotype is not always detected despite T1 presence on western blot.

## Discussion

We have characterized the disease phenotype, PrP^Sc^ gel mobility, and prevalence, of a large cohort of sCJD cases linked to the 129MV genotype. The identification of previously unrecognized histotypes—1C-2PL, 1C-2K, and 2C-PL—makes sCJDMV the most heterogeneous human prion disease [2, 28, 30], and broadens the spectrum of histopathological phenotypes: 1C and 1V described in MV1, and 2C, 2C-K, and 2K identified in MV2 [2, 23]. Although 129MV heterozygosity and disease heterogeneity are coupled in sCJD, it should be emphasized that codon 129MV heterozygous alone does not always lead to phenotypical variability in other human prion diseases [20, 44]. With the exception of 1C, all other histotypes harbor both resPrP^Sc^ types. 1C is the only histotype of MV1, whereas the 1C-2PL, 1C-2K, and 1C-2C histotypes belong to a group of MV1-2 cases with long disease courses and under-representation of T2. The 1C-2C histotype has also been referred to as MV1 + 2C [7]. In MV1-2, T2 is detected in subcortical structures (1C-2PL and 1C-2K), cerebellum (1C-2K), or cerebral cortex (1C-2C). The only case with the 1V histotype harbors a focal 2C component coupled with T2. We believe that in the few cases of 1C histotype with focal cPrP deposits (e.g., cases 7, 8, and 18, Additional file 1: Table S1), resPrP^Sc^ T2 levels may be below the threshold of detectability. This discrepancy may be due to the molecular and histopathologic examination carried out in different cerebral hemispheres [39].

Each histotype of sCJDMV shows a selective regional vulnerability to PrP^Sc^ types resulting in different lesion profiles [18, 26]. In all histotypes, the cerebral cortex and subcortical structures (striatum and thalamus), are most severely affected; the substantia nigra is the least vulnerable of the subcortical structures, allowing replication of PrP^Sc^ from patients with “PL” and “K” histotypic components. The least vulnerable region of the brain is the hippocampus, which is typically affected only in subjects with the 2K and 2C-K histotypes.

In a recent study, we demonstrated a novel mechanism of phenotypic determination [30] which had been previously explored by others [29]. According to our study, in sCJDMV2 preferential conversion of PrP^C^-129 M to PrP^Sc^ is associated with the 2C histotype, whereas a dominant conversion of PrP^C^-129V leads to the 2K histotype. Furthermore, T2 is associated with the 2C histotype in the cerebral cortex, whereas T1^20^-T2 is invariably detected in the 2K histotype (Additional file 1: Table S6) [1]. In patients with the 2C-K histotype, PrP^C^-129M → PrP^Sc^ occurs preferentially in the cerebral cortex, whereas PrP^C^-129V → PrP^Sc^ preferential conversion takes place in the cerebellum [30]. According to this proposition, we believe that the selective vulnerability across the broad spectrum of sCJDMV histotypes, including those reported in this study, is due to the relative ratios of PrP^C^-129M/-129V alleles converted into PrP^Sc^. For example, in cases with the 2C-PL histotype, a “pure T2” is predominantly found in the cerebral cortex, and is rare in subcortical regions. On the contrary, T1^20^-T2 is invariably detected in subcortical regions (Additional file 1: Table S6). Applying this logic to the 2C-PL histotype, we would expect a preferential conversion of (i) PrP^C^-129V into PrP^Sc^ T1^20^-T2 in subcortical regions (harboring plaque-like PrP), and (ii) PrP^C^-129M → PrP^Sc^ T2 in the cerebral cortex. A similar mechanism may be invoked in 1C-2PL and 1C-2K histotypes. In these groups, plaque-like/plaque PrP patterns and T2 co-distribute in subcortical regions and cerebellum, suggesting that PrP^C^-129V → PrP^Sc^ preferential conversion occurs in these regions. We also hypothesize that misfolding and accumulation of a slower replicating PrP^Sc^ T2 may start in subcortical structures, with the faster replicating PrP^Sc^ T1^20^ spreading throughout the brain later in the disease course. This hypothesis is supported by evidence that the disease epicenter is in the subcortical regions and cerebellum in patients with the 2K histotype [37]. Furthermore, in bioassay with transgenic mice expressing the human PrP-129MV, incubation periods are significantly longer for sCJD MV2 than MV1 prions [6, 15, 28]. Specifically, PrP^Sc^ from MV2 of 2K and 2C histotypes show a 2- to 2.3-fold longer incubation period than MV1 (1C histotype) [15]. These data suggest that prions propagate slowly in MV2. However, the preferential conversion of PrP^C^ -129M or -129V into PrP^Sc^, known to be a major determinant of disease phenotype, does not necessarily translate into distinct incubation periods in bioassays experiments [43]. Finally, the presence of medium-size vacuoles and ballooned neurons that typically feature in the VV1 subtype may be due to predominant expression of PrP^C^-129V in the 1V histotype [23].

We have determined histotype prevalences in MV2-1. It should not be surprising that 2C-K is the most common histotype, as the likelihood that both 129 alleles, rather than only one, are converted into PrP^Sc^ is higher. Furthermore, the observation that 2C is the second most common histotype, and 2K the least common, suggests that PrP^C^-129M is preferentially converted into PrP^Sc^ in agreement with the higher prevalence of 2C-K with high burdens of cPrP accumulating in the cerebral cortex. Indeed, the proportion of 2C-K cases with high burden of cPrP exceeds 60%, whereas the proportion of 2C-K cases with a low burden of cPrP is only 27%. Another intriguing observation is the co-occurrence of T1^20^ and T1^21^ in sCJD [4, 11, 14]. T1^20^ is by far the major T1 species in sCJDMV. We suggest that T1^20^ detected in the cerebral cortex and subcortical region/cerebellum have divergent biochemical features in 1C-2K. At pH 6.9, T1 of 1C-2K appears as T1^21^ in the cerebral cortex, and as T1^20^ in the cerebellum. Under the same experimental conditions, only T1^21^ is detected in 1C-2C and 1C histotypes. Furthermore, in patients with the 2K histotype (characterized by a marked and widespread PrP plaque pathology), a dominant T1^21^ is never found. In summary, T1 of 1C and 1C-2C histotypes is sensitive to a change in pH, with a predominant T1^21^ or T1^20^ detected in all brain regions at pH 6.9 or pH 8.0; on the contrary, T1^20^ is the only T1 variant in the cerebellum of 1C-2K regardless of the buffer pH. Another important piece of evidence on the effect of PrP^C^-129M/V to PrP^Sc^ preferential conversion is highlighted by the T1^21^ prevalence across the histotypes of MV2-1. A prominent T1^21^ is never found in 2K histotype, but its prevalence increases progressively from 2C-K to 2C-PL/2C. Furthermore, T1^21^ predominates in the cerebral cortex, the brain region where PrP plaque density is the lowest, and cPrP is the highest. It should also be mentioned that while the prevalence of T1 variants is likely to be driven by 129M/V preferential conversion in sCJDMV, a different mechanism must be involved in sCJDVV and sCJDMM cases with co-existing T1 variants [4, 11, 14].

We found that the burden of cortical cPrP is significantly higher in 2C-K patients with longer disease duration. We observed a similar phenomenon in sCJDMM1-2, and suggested that T2 accumulation would play a major role in determine disease duration [7]. This hypothesis is corroborated by transmission studies in which incubation periods inversely correlate with prion titers [3], and levels of PrP^Sc^ increase over time [42].

We believe that the 1C-2PL histotype does not represent an intermediate, immature form of the 1C-2K histotype. Two major observations support this hypothesis. The first is that 1C-2PL and 1C-2K have virtually identical disease durations. If 1C-2PL was the immature form of 1C-2K, 1C-2PL would be expected to have a significantly shorter disease duration than 1C-2K. The second is that PrP plaques are always found in the 2K histotype regardless of disease duration [1]. The same principle could also apply to the 2C-PL and 2C-K histotypes, showing similar disease durations (14 ± 6 and 14 ± 8 months).

In conclusion, the highly heterogeneous histotypes and the number of co-existing PrP^Sc^ types make sCJDMV the most heterogeneous human prion disease. This heterogeneity is likely to be driven by the tendency of both PrP^C^-129M and -129V polymorphic forms to convert to PrP^Sc^ types 1 variants and type 2 in different proportions. Our results also suggest that T1^20^ of the 2K histotype and T1^20^ affecting the cerebellum in 1C-2K belong to the same prion strain. It is not surprising that 2 of the 3 novel histotypes were identified in MV1-2, as this case-cohort represents the least studied sCJDMV, accounting for ~ 22% of the 177 MV1 cases available at the NPDPSC. In addition to the identification of novel histotypes of sCJD [4], other approaches, such as multi-omics and digital pathology as well as cryo-EM and transmission studies are needed to further understand these complex disorders.

### Supplementary Information

**Additional file 1: Supplementary Materials. Table S1.** Age at onset, disease duration, information on western blot (WB) analysis, histotypic and clinical determination in sCJDMV cases examined in this study. **Table S2.** Histopathological features of sCJD MV1 and MV1-2. **Table S3.** Lesion profiles statistical significance. **Table S4.** Histopathological features of sCJDMV2-1. **Table S5.** resPrP^Sc^ type prevalence. **Table S6.** Distribution of resPrP^Sc^ T1 and T2 in the brain of sCJDMV2-1.

**Additional file 2: Fig. S1.** Histopathology of one case with 1V histotype. **a** and **b** H&E. **c**–**f** PrP immunostaining. **a**, **b** Medium-size (**a**, **b**) and often confluent vacuoles (**b**); inset, **a** a ballooned neuron. **c**, **d** Diffuse PrP. **e** “Brush stroke-like” PrP; Mol. L.: molecular layer; Grl. L.: granular layer. **f** Negative PrP staining of the substantia nigra; antibody: 3F4.

**Additional file 3: Fig. S2.** Comparison of lesion profiles from MV1/MV1-2 and MV2-1 subtypes. **a**–**c** Unlike “C” (**a**) and “C-PL” (**b**) histotypes, 1C-2K and 2C-K histotypes (**c**) show different lesion profiles. *P = 0.01–0.05; ***P = 0.0001–0.001.

**Additional file 4: Fig. S3.** Plaque-like PrP pattern of 2C-PL. **a** Scattered plaque-like PrP deposits in a background of diffuse PrP. **b** A plaque-like PrP; Grl. L: granular layer; Mol. L: molecular layer; antibody: 3F4.

**Additional file 5: Fig. S4.** Disease duration, age at onset, and cortical coarse PrP burden of 2C-K. **a** and **b** Disease duration (**a**), but not age at onset (**b**), positively correlates with the levels of coarse PrP; *P<0.02.

**Additional file 6: Fig. S5.** T2 detectability by tohoku-2 antibody in 1C, 1C-2PL and 1C-2K. Only the un-glycosylated isoform of resPrP^Sc^ is shown for convenience in **a**. Near-infrared, LICOR and 8.7-cm long gel (**a** and **b**). **a** Tohoku-2 immunoreacts with T2 in 1C-2PL and 1C-2K histotypes, but not in 1C; TH: anterior thalamus. **b** 3F4 (green dye) binds to T120 in all brain regions, whereas tohoku-2 (red dye) detects a band of ~ 19 kDa in the caudate nucleus (CN) and in three different areas of the cerebellar hemispheres (CE). FC: frontal cortex; OC: occipital cx.

**Additional file 7: Fig. S6.** Effect of the buffer pH on gel mobility of T1 and T2. Brain homogenates (BH) were prepared with LB100 pH 6.9 and 8.0, digested with PK optima of 32 and 10 U/ml, respectively, and run on a 20 cm-long gel (**a**, **c**–**g**; chemiluminescence) or 8.7-cm long gel (**b**; near-infrared, LICOR). Only the un-glycosylated isoform of resPrP^Sc^ is shown for convenience. **a** T1 appears as either T1^21-20^ at pH 6.9 or T1^20^ at pH 8.0; T2 is made of a single fragment of ~ 19 kDa at both pHs. **b** At buffer pH 6.9, T1 migrates to ~ 21 kDa or ~ 20 kDa depending on whether PrP^Sc^ is harvested from the frontal cortex (FC) or cerebellum (CE). **c** resPrP^Sc^ migrates as a doublet T1^21^-20 at buffer pH 6.9, or as T1^20^ at buffer pH 8.0. T2 co-exists with T1 in alla brain regions. **d** resPrP^Sc^ appears as T1^21-20^-T2 at buffer pH 6.9, or as T1^20^-T2 at buffer pH 8.0; 5 U/ml PK was used in addition to 32 U/ml (pH 6.9) and 10 U/ml (pH 8.0). **e**–**g** Gel mobility of T1^21-20^-T2 (**e**) and T1^20^-T2 (**f** and **g**) is not influenced by the buffer pH; arrowhead, **e** lower resPrP^Sc^ fragment of ~ 18 kDa. FC: frontal cortex; TC: temporal cortex; PC: parietal cortex; ST: striatum; TH: anterior thalamus. T1^21-20^: T1^21^ predominant over T1^20^.

**Additional file 8: Fig. S7.** Typing of resPrP^Sc^ in 1V. Only the un-glycosylated isoform of resPrP^Sc^ is shown for convenience. **a** and **b** Chemiluminescence (**a** and **b**), 8.7- (**a**) and 20-cm (**b**) long gels. **a** Only T1^20^ is detected in a 8.7-cm gel. At longer film exposures, T1^20^ is detected also in the striatum (ST) and substantia nigra (SN) (not shown). **b** In the cerebral cortex T1^20^ co-exists with a slower migrating band of ~ 19 kDa (arrow) matching the gel mobility of T2. FC_s_: frontal cortex (cx), superior gyrus; FC_M_: FC, middle gyrus; TC: temporal cx; PC: parietal cx; HI: hippocampus; EC: entorhinal cx; VC: visual cx; OC: occipital cx; TH: anterior thalamus; CE_1-4_: four distinct regions of the cerebellum.

**Additional file 9: Fig. S8.** Brain distribution of T2 in 1C-2PL, 1C-2K and 1C-2C. **a **and **b** In 1C-2PL and 1C-2K, T2 preferentially accumulates in the subcortical regions (Subc) (**a** and **b**), and to a lesser extent in the cerebellum (Crbl) in 1C-2K (**b**). **c** In 1C-2C, T2 accumulates is similar proportions in each brain compartment; CC: cerebral cortex. *P<0.03-0.05; **P<0.002.

**Additional file 10: Fig. S9.** RT-QuIC of thalamic PrP^Sc^ from 1C-2P and 1C-2K histotypes. **a** and **b** Similar seeding kinetics of PrP^Sc^ are seen at 10^-4^ (**a**) and 10^-6^ (**b**) dilutions. **c** Seeding activity is more efficient at greater dilutions (10^-8^) in 1C-2K. *P<0.05; ^*P* = 0.06; ^$^*P* = 0.08.

## Data Availability

The data supporting the findings of this study are included in tables and supplemental materials.

## Abbreviations

sCJD: Sporadic Creutzfeldt–Jakob disease
T1, T2: Type 1, Type 2
PrP: Prion protein
PrP^C^: Cellular PrP
PrP^Sc^: Scrapie PrP
resPrP^Sc^: Proteinase K (PK)-resistant PrP^Sc^
cPrP: Coarse PrP
T1^21^: T1 w. unglycos. resPrP^Sc^ of ~ 21 kDa
T1^20^: T1 w. unglycos. resPrP^Sc^ of ~ 20 kDa
MV1-2: SCJDMV with mixture of T1 and T2, and T1 > T2
MV2-1: SCJD with mixture of T1 and T2, and T2 > T1
129M: Methionine at codon 129
129V: Valine at codon 129
CSF: Cerebrospinal fluid
PSWCs: Periodic sharp wave complexes (PSWCs) on electroencephalography (EEG)
RT-QuIC: Real-time quaking-induced conversion
recHaPrP: Syrian hamster recombinant PrP

## Histotypes (below)

2C: Cortical (C) cPrP
2K: Kuru (K) plaques
2C-K: Mixed 2C and 2K histotypic features
1C: Cortical (C) diffuse PrP
1C-2PL: 1C and plaque-like (PL) PrP
1C-2K: 1C and kuru (K) plaques
1V: Resembling sCJDVV1
