# Supplementary material for: Novel histotypes of sporadic Creutzfeldt–Jakob disease linked to 129MV genotype
Source: Acta Neuropathol Commun. 2023 Aug 31;11:141. doi: 10.1186/s40478-023-01631-9 (PMC10469800; doi:10.1186/s40478-023-01631-9)
Supplement: Supplementary file 1 — Additional file 1: Supplementary Materials. Table S1. Age at onset, disease duration, information on western blot (WB) analysis, histotypic and clinical determination in sCJDMV cases examined in this study. Table S2. Histopathological features of sCJD MV1 and MV1-2. Table S3. Lesion profiles statistical significance. Table S4. Histopathological features of sCJDMV2-1. Table S5. resPrPSc type prevalence. Table S6. Distribution of resPrPSc T1 and T2 in the brain of sCJDMV2-1. [file 40478_2023_1631_MOESM1_ESM.docx]

**Novel histotypes of sporadic Creutzfeldt-Jakob disease linked to 129MV genotype**

Laura Cracco,^1^ Gianfranco Puoti,^2^* Antonio Cornacchia,^3^* Katie Glisic,^7^ Seong‑Ki Lee,^6^ Zerui Wang,^3^ Mark L. Cohen,^3,7^ Brian S. Appleby,^3,4,5,7^ Ignazio Cali^3,7^

^1^Department of Pathology and Laboratory Medicine, School of Medicine, Indiana University, Indianapolis, IN, United States; ^2^Division of Neurology, University of Campania “Luigi Vanvitelli”, Caserta, Italy; Departments of ^3^Pathology, ^4^Neurology, ^5^Psychiatry, ^6^Physiology and Biophysics, School of Medicine, Case Western Reserve University; ^7^National Prion Disease Pathology Surveillance Center, Cleveland, OH 44106, USA.

*Equal contribution

Corresponding author: Dr. Ignazio Cali

E-mail: [ixc20@case.edu](mailto:ixc20@case.edu)

Tel: 216-368-4874

Fax: 216-368-4090

**SUPPLEMENTARY MATERIALS**

**Reagents and antibodies**

Sodium deoxycholate, Tris-HCl, Nonidet P-40, sodium chloride (NaCl), PK, phenylmethanesulfonyl fluoride (PMSF), and Kodak Biomax MR and XAR films were purchased from Sigma Aldrich (St. Louis, MO, USA); 10X Tris buffered saline (TBS), 10X Dulbecco phosphate buffered saline (DPBS), Tween 20, sodium dodecyl sulfate (SDS), β-mercaptoethanol, 30% Acrylamide/Bis solution, tetramethylethylenediamine, ammonium persulphate, 10% sodium dodecyl sulfate (SDS) from Bio-Rad Laboratories (Hercules, CA, USA); Polyvinylidene Fluoride (PVDF) membrane from Millipore (Bedford, MA, USA); ethylenediaminetetraacetic acid, methanol, guanidine hydrochloride from Promega (Madison, WI, USA). Chemiluminescence substrate (ECL plus) from Amersham Biosciences (Piscataway, NJ, USA). Dako Envision + System HRP Labelled Anti-Mouse, and Envision Flex DAB from Agilent (Santa Clara, CA). The Odyssey blocking buffer, the Infrared Dye (IRDye) 800CW goat anti-mouse IgG (1 mg/ml) and IRDye 680RD goat anti-rabbit IgG (1 mg/ml), as well as the Polyvinylidene difluoride (PVDF) membrane (Immobilon-FL) were from LICOR Biosciences (Lincoln, NE, USA). Thioflavin T, and N-2 Supplement were from Thermofisher (Waltham, MA, USA). Antibodies used were: 3F4 to human PrP (HuPrP) residues 106-110 [1], 1E4 to HuPrP residues 97-108 (Wageningen University & Research; Lelystad, Netherlands) [2], and Tohoku 2 to HuPrP residues 97–103 (kindly provided by Dr. Tetsuyuki Kitamoto) [3].

[1] Kascsak RJ, Rubenstein R, Merz PA, Tonna-DeMasi M, Fersko R, Carp RI, Wisniewski HM, Diringer H (1987) Mouse polyclonal and monoclonal antibody to scrapie-associated fibril proteins. J Virol 61:3688–3693

[2] Langeveld JPM, Jacobs JG, Erkens JHF, Bossers A, van Zijderveld FG, van Keulen LJM (2006) Rapid and discriminatory diagnosis of scrapie and BSE in retro-pharyngeal lymph nodes of sheep. BMC Vet Res 2:19

[3] Kobayashi A, Sakuma N, Matsuura Y, Mohri S, Aguzzi A, Kitamoto T (2010) Experimental verification of a traceback phenomenon in prion infection. J Virol 84:3230–3238

| **Table S1** Age at onset, disease duration, information on western blot (WB) analysis, histotypic and clinical determination in sCJDMV cases examined in this study | | | | | | | | | | |
| --- | --- | --- | --- | --- | --- | --- | --- | --- | --- | --- |
| Case n | Age onset  (years) | Disease duration  (months) | Extensive  WB analysis ^a^ | Histotypic & clinical evaluation ^b^ |  | Case n | Age onset  (years) | Disease duration  (months) | Extensive  WB analysis ^a^ | Histotypic & clinical evaluation ^b^ |
| 1 | 73 | 3 | + | +/+ |  | 56 | 78 | 23 | + | +/+ |
| 2 | 81 | 1.5 | + | +/+ |  | 57 | 79 | 10 | + | +/+ |
| 3 | 75 | 4 | + | +/+ |  | 58 | 66 | 9 | – | +/na |
| 4 | 74 | 4 | + | +/+ |  | 59 | 73 | 11 | – | +/+ |
| 5 | 74 | 2.5 | + | +/+ |  | 60 | 64 | 17 | + | +/+ |
| 6 | 53 | 4 | + | +/+ |  | 61 | 76 | 13 | + | +/+ |
| 7 | 78 | 2 | + | +/+ |  | 62 | 71 | 11 | + | +/+ |
| 8 | 71 | 3 | + | +/+ |  | 63 | 81 | 24 | + | +/+ |
| 9 | 47 | 3 | + | +/+ |  | 64 | 65 | 9 | + | +/+ |
| 10 | 72 | 2 | + | +/+ |  | 65 | 73 | 9 | + | +/+ |
| 11 | 48 | 3 | + | +/+ |  | 66 | 73 | 6 | + | +/+ |
| 12 | 79 | 4 | + | +/+ |  | 67 | 63 | 3 | + | +/+ |
| 13 | 72 | 3.5 | + | +/+ |  | 68 | 66 | 14 | + | +/+ |
| 14 | 68 | 3 | + | +/+ |  | 69 | na | na | + | +/+ |
| 15 | 68 | 4 | + | +/+ |  | 70 | 58 | 27 | + | +/+ |
| 16 | 65 | 3 | + | +/+ |  | 71 | 77 | 10 | + | +/+ |
| 17 | 84 | 18 | – | +/+ |  | 72 | na | na | + | +/+ |
| 18 | 54 | 27 | + | +/+ |  | 73 | 58 | 23 | + | +/+ |
| 19 | 67 | 20 | – | +/+ |  | 74 | 68 | 26 | – | +/– |
| 20 | 65 | 15 | – | +/+ |  | 75 | 74 | 6 | – | +/– |
| 21 | 80 | 12 | – | +/+ |  | 76 | 78 | 15 | – | +/– |
| 22 | 64 | 31 | + | +/+ |  | 77 | 65 | 12 | – | +/– |
| 23 | 76 | 17 | + | +/+ |  | 78 | 72 | 11 | – | +/– |
| 24 | 68 | 16 | + | +/+ |  | 79 | 71 | 12 | – | +/– |
| 25 | 77 | 4 | + | +/+ |  | 80 | 67 | 8 | – | +/– |
| 26 | 66 | 10 | + | +/+ |  | 81 | 68 | 9 | – | +/– |
| 27 | 71 | 11 | + | +/+ |  | 82 | 70 | 43 | – | +/– |
| 28 | 74 | 10 | + | +/+ |  | 83 | 67 | 8 | – | +/– |
| 29 | 74 | 15 | + | +/+ |  | 84 | 46 | 14 | – | +/– |
| 30 | 72 | 4 | + | +/+ |  | 85 | 69 | 35 | – | +/– |
| 31 | 70 | 10 | + | +/+ |  | 86 | 64 | 6 | – | +/– |
| 32 | 67 | 46 | + | +/+ |  | 87 | 79 | 33 | – | +/– |
| 33 | 82 | 22 | + | +/+ |  | 88 | 64 | 9 | – | +/– |
| 34 | 60 | 16 | + | +/+ |  | 89 | 76 | 4 | – | +/– |
| 35 | 50 | 32 | + | +/+ |  | 90 | 87 | 13 | – | +/– |
| 36 | 71 | 58 | – | +/+ |  | 91 | 66 | 10 | – | +/– |
| 37 | 70 | 25 | – | +/+ |  | 92 | 65 | 6 | – | +/– |
| 38 | 63 | 42 | – | +/+ |  | 93 | 56 | 14 | – | +/– |
| 39 | 78 | 4 | – | +/+ |  | 94 | 81 | 8 | – | +/– |
| 40 | 63 | 30 | – | +/+ |  | 95 | 66 | 7 | – | +/– |
| 41 | 79 | 24 | – | +/na |  | 96 | 63 | 16 | – | +/– |
| 42 | 79 | 19 | – | +/+ |  | 97 | 77 | 3 | – | +/– |
| 43 | 75 | 13 | – | +/+ |  | 98 | 68 | 7 | – | +/– |
| 44 | 57 | 17 | – | +/+ |  | 99 | 72 | 7 | – | +/– |
| 45 | 66 | 42 | – | +/+ |  | 100 | 73 | 9 | + | +/+ |
| 46 | 77 | 8 | – | +/na |  | 101 | 74 | 19 | + | +/+ |
| 47 | 62 | 45 | – | +/+ |  | 102 | 79 | 8 | + | +/+ |
| 48 | 77 | 8 | – | +/+ |  | 103 | 56 | 28 | + | +/+ |
| 49 | 75 | 69 | – | +/+ |  | 104 | 63 | 9 | + | +/+ |
| 50 | 59 | 21 | – | +/+ |  | 105 | 53 | 18 | + | +/+ |
| 51 | 82 | 15 | – | +/+ |  | 106 | 69 | 7 | + | +/+ |
| 52 | 72 | 20 | – | +/+ |  | 107 | 68 | 16 | + | +/+ |
| 53 | 61 | 25 | – | +/+ |  | 108 | 63 | 12.5 | + | +/+ |
| 54 | 77 | 22 | – | +/+ |  | 109 | 66 | 9 | + | +/+ |
| 55 | 62 | 16 | + | +/+ |  | 110 | 68 | 19 | + | +/– |
| Cases 1-31 and 32-100 were diagnosed at the NPDPSC (by routine WB analysis of 3 brain regions) as sCJD MV1 and MV2, respectively. ^a^ Six or more brain regions assessed by western blot; ^b^ Histotype only (+/–) or histotype and clinical history (+/+) assessed. | | | | | | | | | | |

| **Table S2** Histopathological features of sCJD MV1 and MV1-2 | | | | | | | | | | | | | | | | | | | | | | | | | |
| --- | --- | --- | --- | --- | --- | --- | --- | --- | --- | --- | --- | --- | --- | --- | --- | --- | --- | --- | --- | --- | --- | --- | --- | --- | --- |
| Histotype | Case n | Age at onset  (years) | Disease duration  (months) | Vacuole size | | |  | PrP immunostaining pattern | | | | | | | | | | | | | | | | | |
|  |  |  |  | Medium |  | Large |  | Coarse ^a^ |  | Plaque-like | | | | | | | | |  | Plaque |  | Brush stroke-like |  | Coarse | |
|  |  |  |  | CC+ST |  | CC ^b^ |  | CC ^b^ |  | CC ^b^ |  | SN |  | ST |  | TH |  | CE |  | CE |  | CE |  | CE | |
| 1C | 1 | 73 | 3 | – |  | – |  | 0 ^c^ |  | – |  | – |  | – |  | – |  | – |  | – |  | + |  | – | |
|  | 2 | 81 | 1.5 | – |  | – |  | 0 |  | – |  | – |  | – |  | – |  | – |  | – |  | + |  | – | |
|  | 3 | 75 | 4 | – |  | – |  | 0 |  | – |  | – |  | – |  | – |  | – |  | – |  | + |  | – | |
|  | 4 | 74 | 4 | – |  | – |  | 0 |  | – |  | – |  | – |  | – |  | – |  | – |  | + |  | – | |
|  | 5 | 74 | 2.5 | – |  | – |  | na |  | na |  | na |  | na |  | na |  | na |  | na |  | na |  | na | |
|  | 6 | 53 | 4 | – |  | – |  | 0 |  | – |  | – |  | – |  | – |  | – |  | – |  | + |  | – | |
|  | 7 | 78 | 2 | – |  | – |  | <1 ^d^ |  | – |  | – |  | – |  | – |  | – |  | – |  | + |  | – | |
|  | 8 | 71 | 3 | – |  | – |  | <1 ^d^ |  | – |  | – |  | – |  | – |  | – |  | – |  | + |  | – | |
|  | 9 | 47 | 3 | – |  | – |  | 0 |  | – |  | – |  | – |  | – |  | – |  | – |  | + |  | – | |
|  | 10 | 72 | 2 | – |  | – |  | 0 |  | – |  | – |  | – |  | – |  | – |  | – |  | + |  | – | |
|  | 11 | 48 | 3 | – |  | – |  | 0 |  | – |  | – |  | – |  | – |  | – |  | – |  | + |  | – | |
|  | 12 | 79 | 4 | – |  | – |  | 0 |  | – |  | – |  | – |  | – |  | – |  | – |  | + |  | – | |
|  | 13 | 72 | 3.5 | – |  | – |  | 0 |  | – |  | – |  | – |  | – |  | – |  | – |  | + |  | – | |
|  | 14 | 68 | 3 | – |  | – |  | 0 |  | – |  | – |  | – |  | – |  | – |  | – |  | + |  | – | |
|  | 15 | 68 | 4 | – |  | – |  | 0 |  | – |  | – |  | – |  | – |  | – |  | – |  | + |  | – | |
|  | 16 | 65 | 3 | – |  | – |  | 0 |  | – |  | – |  | – |  | – |  | – |  | – |  | + |  | – | |
|  | 17 | 84 | 18 | – |  | – |  | 0 |  | – |  | – |  | – |  | – |  | – |  | – |  | + |  | – | |
|  | 18 | 54 | 27 | – |  | – |  | <1 |  | – |  | – |  | – |  | – |  | – |  | – |  | + |  | – | |
|  | 19 | 67 | 20 | +/– |  | – |  | 0 |  | – |  | – |  | – |  | – |  | – |  | – |  | + |  | – | |
|  | 20 | 65 | 15 | +/– |  | – |  | 0 |  | – |  | – |  | – |  | – |  | – |  | – |  | + |  | – | |
|  | 21 | 80 | 12 | – |  | – |  | 0 |  | – |  | – |  | – |  | – |  | – |  | – |  | + |  | – | |
| 1V | 22 | 64 | 31 | + |  | – |  | <1 |  | – |  | – |  | – |  | – |  | – |  | – |  | + |  | – | |
| 1C-2PL | 23 | 76 | 17 | – |  | – |  | 0 |  | – |  | + |  | + |  | na |  | – |  | – |  | + |  | – | |
|  | 24 | 68 | 16 | – |  | – |  | <1 |  | – |  | + |  | – |  | na |  | + r |  | – |  | – |  | – | |
|  | 25 | 77 | 4 | +/– |  | – |  | <1 |  | – |  | + |  | + |  | + |  | – |  | – |  | + |  | – | |
| 1C-2K | 26 | 66 | 10 | +/– |  | – |  | <1 |  | – |  | + |  | + |  | na |  | + |  | + |  | + |  | – | |
|  | 27 | 71 | 11 | +/– |  | – |  | 0 |  | – |  | + |  | + |  | na |  | + |  | + |  | + |  | na | |
|  | 28 | 74 | 10 | – |  | – |  | <1 |  | + |  | + |  | + |  | na |  | + |  | + |  | + |  | – | |
|  | 29 | 72 | 15 | – |  | + |  | 3 |  | – |  | + |  | + |  | + |  | + |  | + |  | + |  | – | |
| 1C-2C | 30 | 72 | 4 | – |  | + |  | 5 |  | – |  | – |  | – |  | – |  | – |  | – |  | + |  | – | |
|  | 31 | 70 | 10 | – |  | + |  | 15 |  | – |  | – |  | – |  | – |  | – |  | – |  | + |  | – | |
| ^a^ Focal or patchy deposits. ^b^ It includes frontal, occipital, temporal and parietal cortices. ^c^ Percentage of surface of the cerebral cortex occupied by coarse PrP. ^d^ Cases with rare coarse PrP deposits in the frontal (case 8) or occipital (case 7) cortex, and in absence of large vacuoles. r: rare. Ballooned neurons were found in case 22. CC: Cerebral cortex; ST: striatum; TH: anterior thalamus; SN: substantia nigra; CE: cerebellum; Grl. L: granular layer; Mol. L: molecular layer; na: not available. | | | | | | | | | | | | | | | | | | | | | | | | |  |

| **Table S3** Lesion profiles statistical significance | | | | |
| --- | --- | --- | --- | --- |
| Histotype | 1C short d. | 1C long d. | 1C-2PL | 1C-2K |
| 1C short d. |  | OC ** | HI *; SN ****; TH * | OC *; SN ****; TH * |
| 1C long d. |  |  | SN **** | SN ****; TH * |
| 1C-2PL |  |  |  |  |
| 1C-2K |  |  |  |  |
|  | 2C | 2C-PL | 2C-K | 2K |
| 2C |  | SN **** | OC<0.02; HI ***; SN ****; ST *; TH * | OC *; HI ****; SN ****; ST *; TH *; CE *** |
| 2C-PL |  |  | HI ** | HI ***; SN **; CE ** |
| 2C-K |  |  |  | SN *; CE *** |
| 2K |  |  |  |  |
| OC: occipital cortex; HI: hippocampus (CA1-CA4); SN: substantia nigra; TH: thalamus; ST: striatum; CE: cerebellum; short and long d.: short and long disease duration; **P*=0.01-0.05; ***P*=0.001-0.01; ****P*=0.0001-0.001; *****P*<0.0001. One-way ANOVA | | | | |
|  | | | | |

| **Table S4** Histopathological features of sCJDMV2-1 | | | | | | | | | | | | | | |
| --- | --- | --- | --- | --- | --- | --- | --- | --- | --- | --- | --- | --- | --- | --- |
| Histotype | Case n |  | PrP immunostaining pattern | | | | | | |  | 1C histotype |  | Prominent T1^21 c^ |  |
|  |  |  | Coarse ^a^ | | |  | Plaque |  | Plaque-like |  |  |  |  |  |
|  |  |  | CC ^b^ |  | CE |  | CE |  | Brain |  |  |  | Brain |  |
| 2C | 32 |  | 75 |  | + |  | – |  | – |  | – |  | – |  |
|  | 33 |  | 95 |  | + |  | – |  | – |  | – |  | – |  |
|  | 34 |  | 100 |  | + |  | – |  | – |  | + CE |  | + |  |
|  | 35 |  | 100 |  | + |  | – |  | – |  | – |  | – |  |
|  | 36 |  | 100 |  | + |  | – |  | – |  | – |  | na |  |
|  | 37 |  | 100 |  | + |  | – |  | – |  | – |  | na |  |
|  | 38 |  | 100 |  | + |  | – |  | – |  | – |  | na |  |
|  | 39 |  | 40 |  | – |  | – |  | – |  | – |  | na |  |
|  | 40 |  | 100 |  | + |  | – |  | – |  | – |  | na |  |
|  | 41 |  | 100 |  | + |  | – |  | – |  | – |  | na |  |
|  | 42 |  | 100 |  | + |  | – |  | – |  | – |  | na |  |
|  | 43 |  | 100 |  | + |  | – |  | – |  | – |  | na |  |
|  | 44 |  | 90 |  | + |  | – |  | – |  | – |  | na |  |
|  | 45 |  | 100 |  | + |  | – |  | – |  | – |  | na |  |
|  | 46 |  | 70 |  | + |  | – |  | – |  | – |  | na |  |
|  | 47 |  | 100 |  | + |  | – |  | – |  | – |  | na |  |
|  | 48 |  | 60 |  | + |  | – |  | – |  | + OC, r |  | na |  |
|  | 49 |  | 80 |  | + |  | – |  | – |  | – |  | na |  |
|  | 50 |  | 100 |  | + |  | – |  | – |  | – |  | na |  |
|  | 51 |  | 100 |  | – |  | – |  | – |  | – |  | na |  |
|  | 52 |  | 100 |  | + |  | – |  | – |  | – |  | na |  |
|  | 53 |  | 100 |  | + |  | – |  | – |  | – |  | na |  |
|  | 54 |  | 90 |  | + |  | – |  | **–** |  | – |  | na |  |
|  | 32-54 |  | 91±16 ^d^ |  | 91 ^e^ |  | 0 ^e^ |  | 0 ^e^ |  | 9 ^e^ |  | 25 |  |
| 2C-PL | 55 |  | 50 |  | + |  | – |  | + SN,TH |  | + CE |  | + |  |
|  | 56 |  | 100 |  | + |  | – |  | + SN, CE |  | – |  | – |  |
|  | 57 |  | 100 |  | + |  | – |  | + SN, ST |  | – |  | + |  |
|  | 58 |  | 60 |  | – |  | – |  | + SN, ST |  | + (OC, ST, r) |  | na |  |
|  | 59 |  | 100 |  | + |  | – |  | + SN |  | – |  | na |  |
|  | 55-59 |  | 82±25 |  | 80 |  | 0 |  | 100 |  | 40 |  | 67 |  |
| 2C-K | 60 |  | 1 |  | – |  | + |  | + |  | – |  | – |  |
|  | 61 |  | 1 |  | – |  | + |  | + |  | – |  | – |  |
|  | 62 |  | 2 |  | – |  | + |  | + |  | – |  | – |  |
|  | 63 |  | 15 |  | – |  | + |  | + |  | – |  | – |  |
|  | 64 |  | 15 |  | – |  | + |  | + |  | + CC |  | + |  |
|  | 65 |  | 30 |  | + |  | + |  | + |  | – |  | – |  |
|  | 66 |  | 55 |  | + |  | + |  | + |  | – |  | – |  |
|  | 67 |  | 55 |  | + |  | + |  | + |  | – |  | + |  |
|  | 68 |  | 85 |  | + |  | + |  | + |  | – |  | – |  |
|  | 69 |  | 85 |  | + |  | + |  | + |  | – |  | – |  |
|  | 70 |  | 90 |  | + |  | + |  | + |  | – |  | – |  |
|  | 71 |  | 90 |  | + |  | + |  | + |  | – |  | – |  |
|  | 72 |  | 95 |  | + |  | + |  | + |  | – |  | + |  |
|  | 73 |  | 95 |  | + |  | + |  | + |  | – |  | + |  |
|  | 74 |  | 100 |  | + |  | + |  | + |  | – |  | na |  |
|  | 75 |  | 50 |  | + |  | + |  | + |  | – |  | na |  |
|  | 76 |  | 100 |  | – |  | + |  | + |  | – |  | na |  |
|  | 77 |  | 100 |  | + |  | + |  | + |  | – |  | na |  |
|  | 78 |  | 100 |  | + |  | + |  | + |  | + TC |  | na |  |
|  | 79 |  | 100 |  | + |  | + |  | + |  | – |  | na |  |
|  | 80 |  | 100 |  | + |  | + |  | + |  | – |  | na |  |
|  | 81 |  | 100 |  | + |  | + |  | + |  | – |  | na |  |
|  | 82 |  | 100 |  | + |  | + |  | + |  | – |  | na |  |
|  | 83 |  | 80 |  | + |  | + |  | + |  | – |  | na |  |
|  | 84 |  | 70 |  | – |  | + |  | + |  | – |  | na |  |
|  | 85 |  | 60 |  | + |  | + |  | + |  | + FC |  | na |  |
|  | 86 |  | 50 |  | + |  | + |  | + |  | – |  | na |  |
|  | 87 |  | 50 |  | + |  | + |  | + |  | – |  | na |  |
|  | 88 |  | 50 |  | – |  | + |  | + |  | – |  | na |  |
|  | 89 |  | 50 |  | + |  | + |  | + |  | – |  | na |  |
|  | 90 |  | 60 |  | – |  | + |  | + |  | – |  | na |  |
|  | 91 |  | 40 |  | – |  | + |  | + |  | + CC |  | na |  |
|  | 92 |  | 40 |  | – |  | + |  | + |  | – |  | na |  |
|  | 93 |  | 30 |  | – |  | + |  | + |  | – |  | na |  |
|  | 94 |  | 15 |  | – |  | + |  | + |  | – |  | na |  |
|  | 95 |  | 10 |  | + |  | + |  | + |  | + OC, CE |  | na |  |
|  | 96 |  | 10 |  | – |  | + |  | + |  | + CC |  | na |  |
|  | 97 |  | 10 |  | – |  | + |  | + |  | – |  | na |  |
|  | 98 |  | 5 |  | – |  | + |  | + |  | – |  | na |  |
|  | 99 |  | 5 |  | – |  | + |  | + |  | – |  | na |  |
|  | 60-99 |  | 55±36 |  | 57 |  | 100 |  | 100 |  | 15 |  | 28 |  |
| 2K | 100-110 |  | 0 |  | 0 |  | 100 |  | 100 |  | 0 |  | 0 |  |
| ^a^ It includes perivacuolar PrP; ^b^ It refers to frontal, occipital, temporal (FC, OC, TC) and parietal cortices. ^c^ T1^21^>T1^20^. ^d, e^ Expressed as ^d^ mean±SD or ^e^ percentage. CC: cerebral cortex; ST: striatum; CE: cerebellum; TH: anterior thalamus; SN: substantia nigra; r: rare. TH was not stained for PrP in cases 58 and 59. | | | | | | | | | | | | | | |

| **Table S5** resPrP^Sc^ type prevalence | | | | |
| --- | --- | --- | --- | --- |
| Histotype | Case n | T1^21^ | T1^20^-T2 | T1^20^ |
| 1C | 1-16 | – | – | + |
| 1V | 22 | – | + ^a^ | – |
| 1C-2PL | 23-25 | – | + | – |
| 1C-2K | 26-29 | – | + | – |
| 1C-2C | 30, 31 | – | + | – |
| 2C | 32 | – | + | – |
|  | 33 | – | + | – |
|  | 34 | + | + | – |
|  | 35 | – | – | – |
| 2C-PL | 55 | + | + | + |
|  | 56 | – | + | – |
|  | 57 | + | + | – |
| 2C-K | 60 | – | + | – |
|  | 61 | – | + | – |
|  | 62 | – | + | – |
|  | 63 | – | + | – |
|  | 64 | + | + | – |
|  | 65 | – | + | – |
|  | 66 | – | + | – |
|  | 67 | + | + | – |
|  | 68 | – | + | – |
|  | 69 | – | + | – |
|  | 70 | – | + | – |
|  | 71 | – | + | – |
|  | 72 | + | + | – |
|  | 73 | + | + | – |
| 2K | 100-110 | – | + | – |
| ^a^ T2 was detected by 1E4, but not with 3F4. T1^21^: T1^21^>T1^20^ in one or more brain regions. T1^20^-T2: Co-existing resPrP^Sc^ fragments of ~ 19 and ~ 20 kDa. T1^20^: T1 variant detected in absence of T2 in all brain regions assessed | | | | |

| **Table S6** Distribution of resPrP^Sc^ T1 and T2 in the brain of sCJDMV2-1 | | | | | |
| --- | --- | --- | --- | --- | --- |
| Histotype | N cases | resPrP^Sc^  type | Cerebral cortex | Subcortical  regions | Cerebellum |
| 2C | 1 | T2 | 100 | 100 | 100 |
|  |  | T1^21^>T1^20^ | 100 ^a^ (1/1) ^b^ | 50 (2/4) | na |
|  | 3 | T2>T1 ^c^ | 0 (0/1) | 50 (2/4) | na |
|  |  | T2 ^d^ | 94 (15/16) | 43 (3/7) | na |
| 2C-PL |  | T1^21^>T1^20^ | 50 (2/4) | 67 (4/6) | 0 (0/1) |
|  | 3 | T2>T1 | 0 (0/3) | 33 (1/3) | 0 (0/1) |
|  |  | T2 | 75 (12/16) | 14 (1/7) | 0 (0/1) |
| 2C-K |  | T1^21^>T1^20^ | 29 (12/41) | 8 (3/39) | 0 (0/9) |
|  | 14 | T2>T1 | 83 (34/41) | 38 (15/39) | 33 (3/9) |
|  |  | T2 | 27 (23/86) | 0 (0/50) | 12 (2/16) |
| 2K ^e^ |  | T1^21^>T1^20^ | 0 | 0 | 0 |
|  | 5 | T2>T1 | 82 (18/22) | 18 (2/11) | 80 (4/5) |
|  |  | T2 | 0 | 0 | 0 |
| ^a^ Expressed in percentage; ^b^ number of brain regions with the feature listed/total number of brain regions examined; ^c^ T1: T1^21^+T1^20^; ^d^ “pure T2”: T1 is not detected; ^e^ It refers to cases 100-104 of Table S1; resPrP^Sc^: PK-resistant PrP^Sc^. | | | | | |
