# Supplementary figures and images for: Novel histotypes of sporadic Creutzfeldt–Jakob disease linked to 129MV genotype
Source: Acta Neuropathol Commun. 2023 Aug 31;11:141. doi: 10.1186/s40478-023-01631-9 (PMC10469800; doi:10.1186/s40478-023-01631-9)

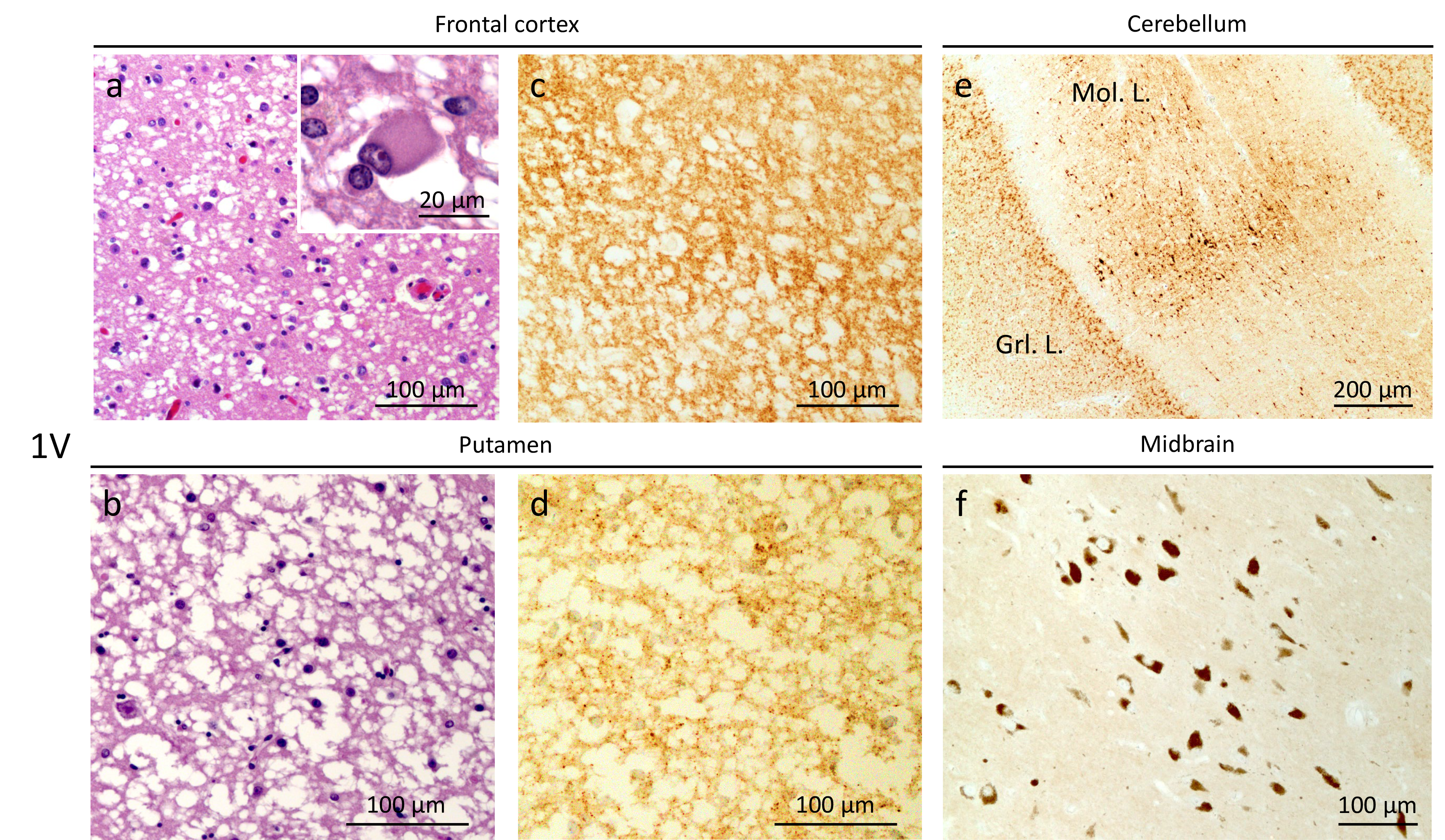

Supplement: Supplementary file 2 — Additional file 2: Fig. S1. Histopathology of one case with 1V histotype. a and b H&E. c–f PrP immunostaining. a, b Medium-size (a, b) and often confluent vacuoles (b); inset, a a ballooned neuron. c, d Diffuse PrP. e “Brush stroke-like” PrP; Mol. L.: molecular layer; Grl. L.: granular layer. f Negative PrP staining of the substantia nigra; antibody: 3F4. [file 40478_2023_1631_MOESM2_ESM.tif]

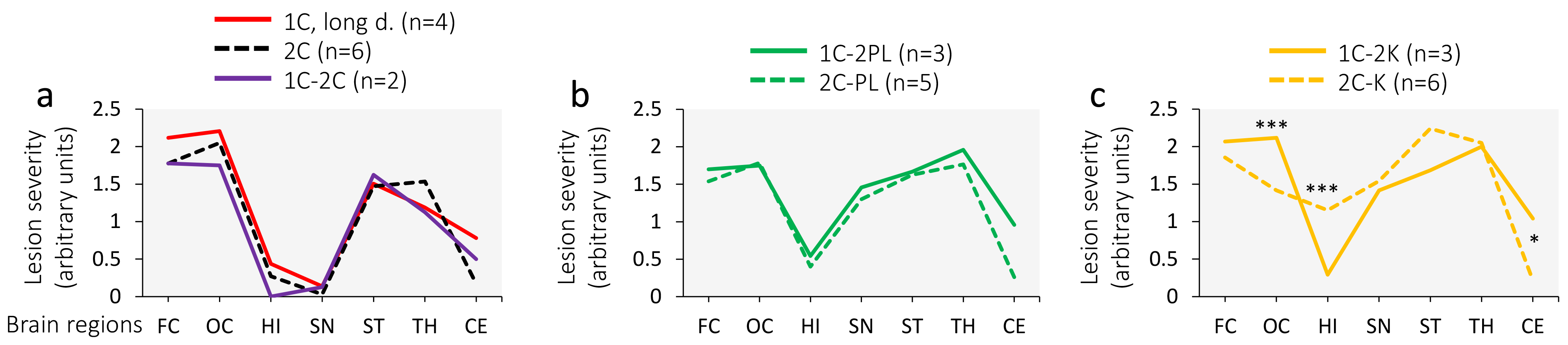

Supplement: Supplementary file 3 — Additional file 3: Fig. S2. Comparison of lesion profiles from MV1/MV1-2 and MV2-1 subtypes. a–c Unlike “C” (a) and “C-PL” (b) histotypes, 1C-2K and 2C-K histotypes (c) show different lesion profiles. *P = 0.01–0.05; ***P = 0.0001–0.001. [file 40478_2023_1631_MOESM3_ESM.tif]

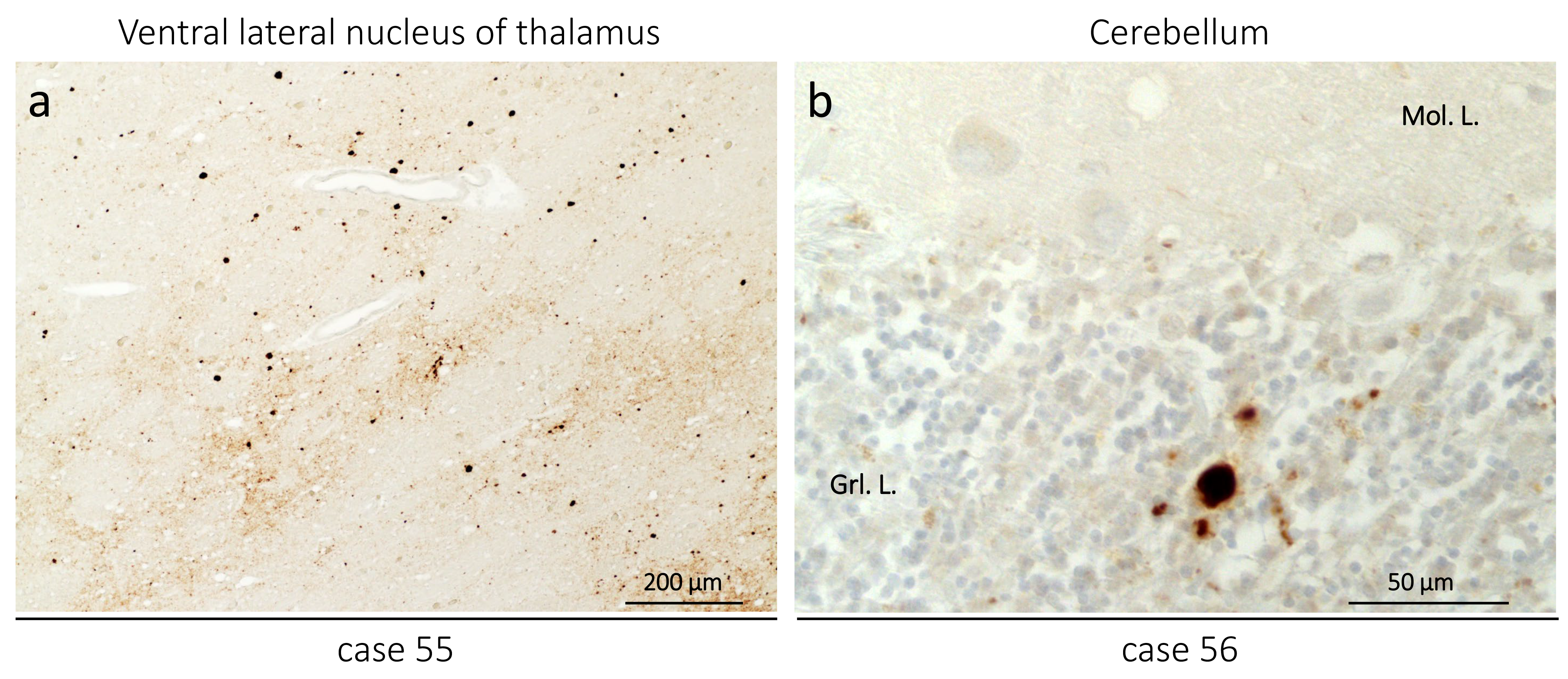

Supplement: Supplementary file 4 — Additional file 4: Fig. S3. Plaque-like PrP pattern of 2C-PL. a Scattered plaque-like PrP deposits in a background of diffuse PrP. b A plaque-like PrP; Grl. L: granular layer; Mol. L: molecular layer; antibody: 3F4. [file 40478_2023_1631_MOESM4_ESM.tif]

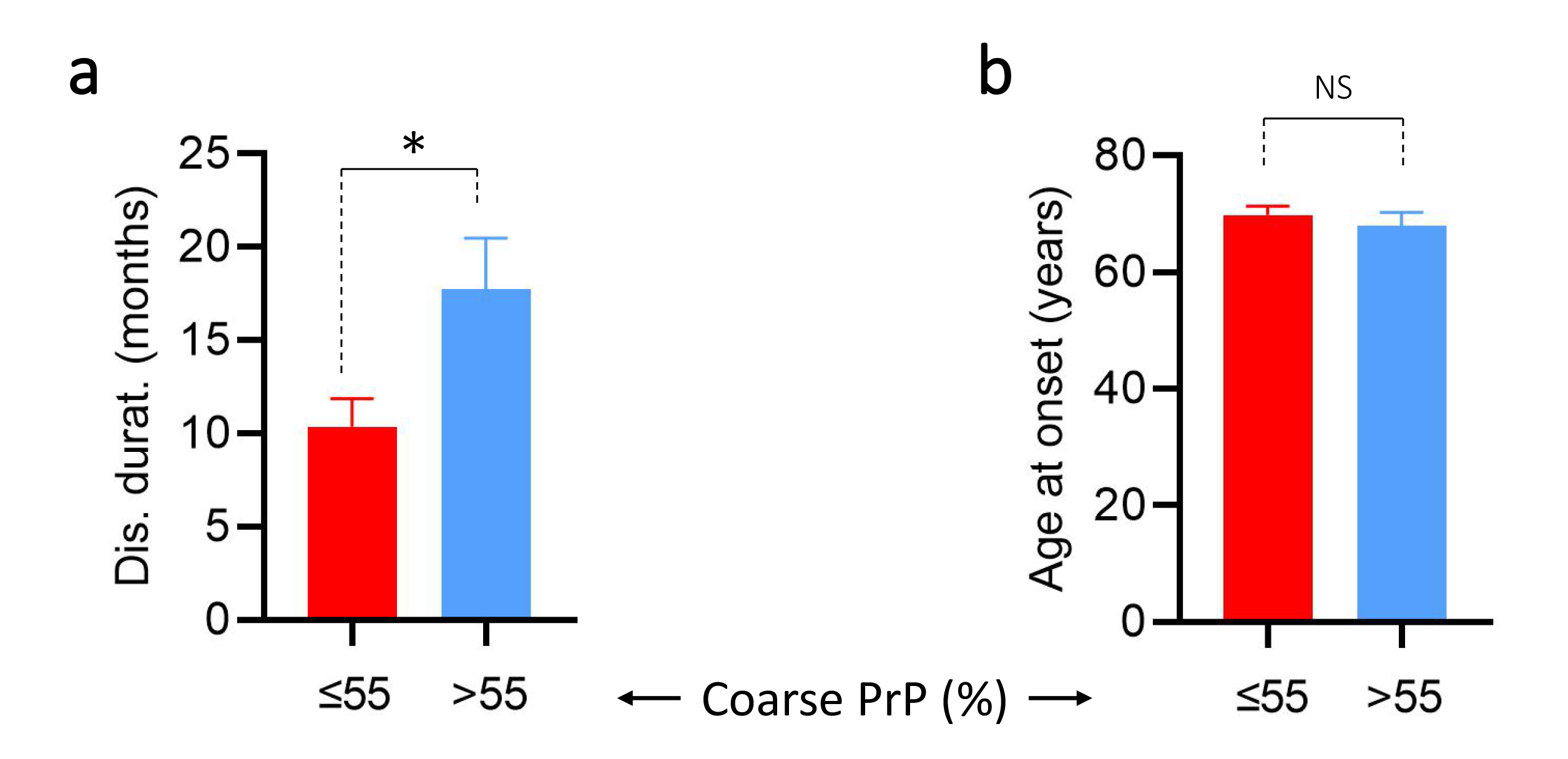

Supplement: Supplementary file 5 — Additional file 5: Fig. S4. Disease duration, age at onset, and cortical coarse PrP burden of 2C-K. a and b Disease duration (a), but not age at onset (b), positively correlates with the levels of coarse PrP; *P<0.02. [file 40478_2023_1631_MOESM5_ESM.tif]

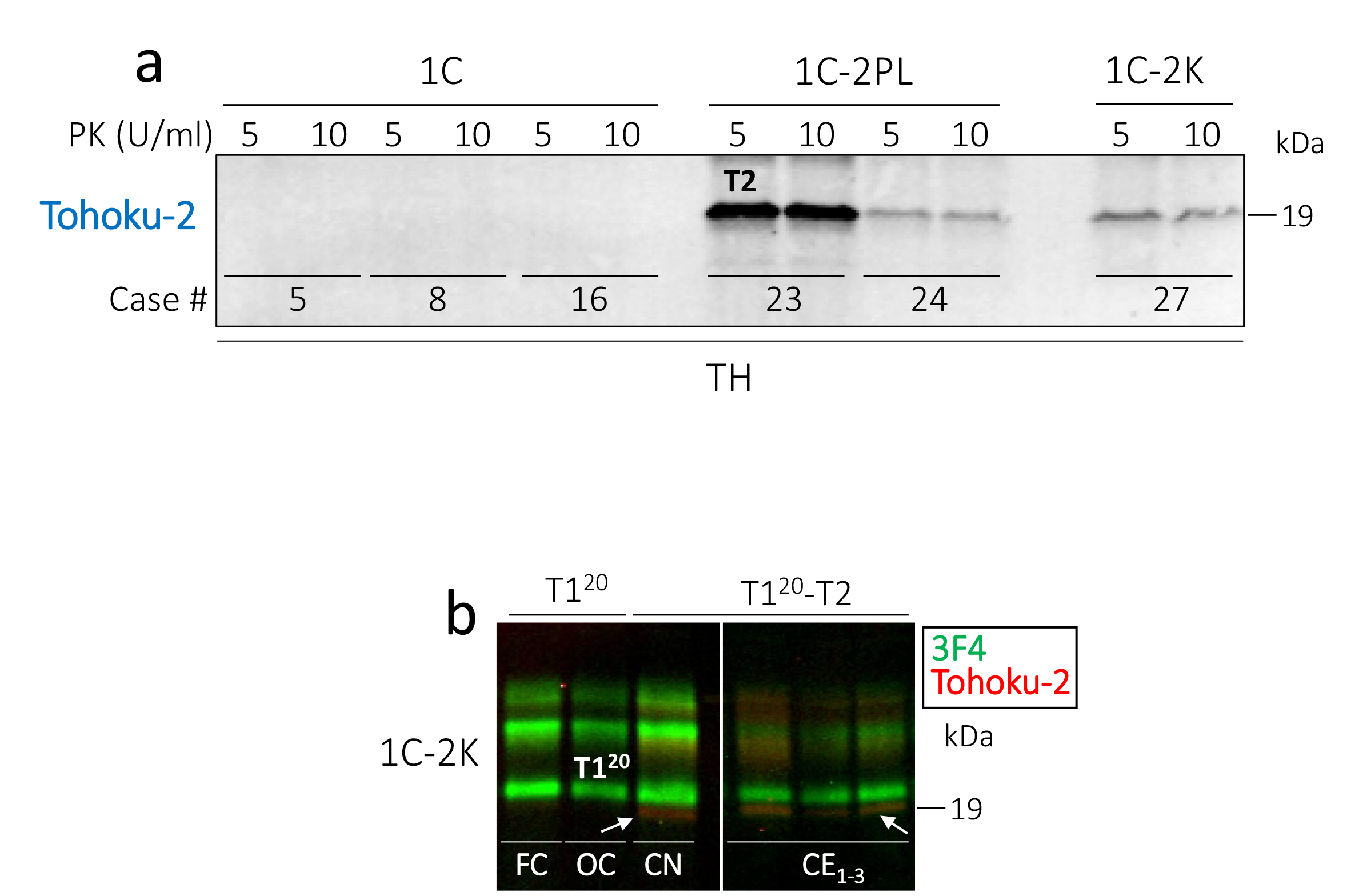

Supplement: Supplementary file 6 — Additional file 6: Fig. S5. T2 detectability by tohoku-2 antibody in 1C, 1C-2PL and 1C-2K. Only the un-glycosylated isoform of resPrPSc is shown for convenience in a. Near-infrared, LICOR and 8.7-cm long gel (a and b). a Tohoku-2 immunoreacts with T2 in 1C-2PL and 1C-2K histotypes, but not in 1C; TH: anterior thalamus. b 3F4 (green dye) binds to T120 in all brain regions, whereas tohoku-2 (red dye) detects a band of ~ 19 kDa in the caudate nucleus (CN) and in three different areas of the cerebellar hemispheres (CE). FC: frontal cortex; OC: occipital cx. [file 40478_2023_1631_MOESM6_ESM.tif]

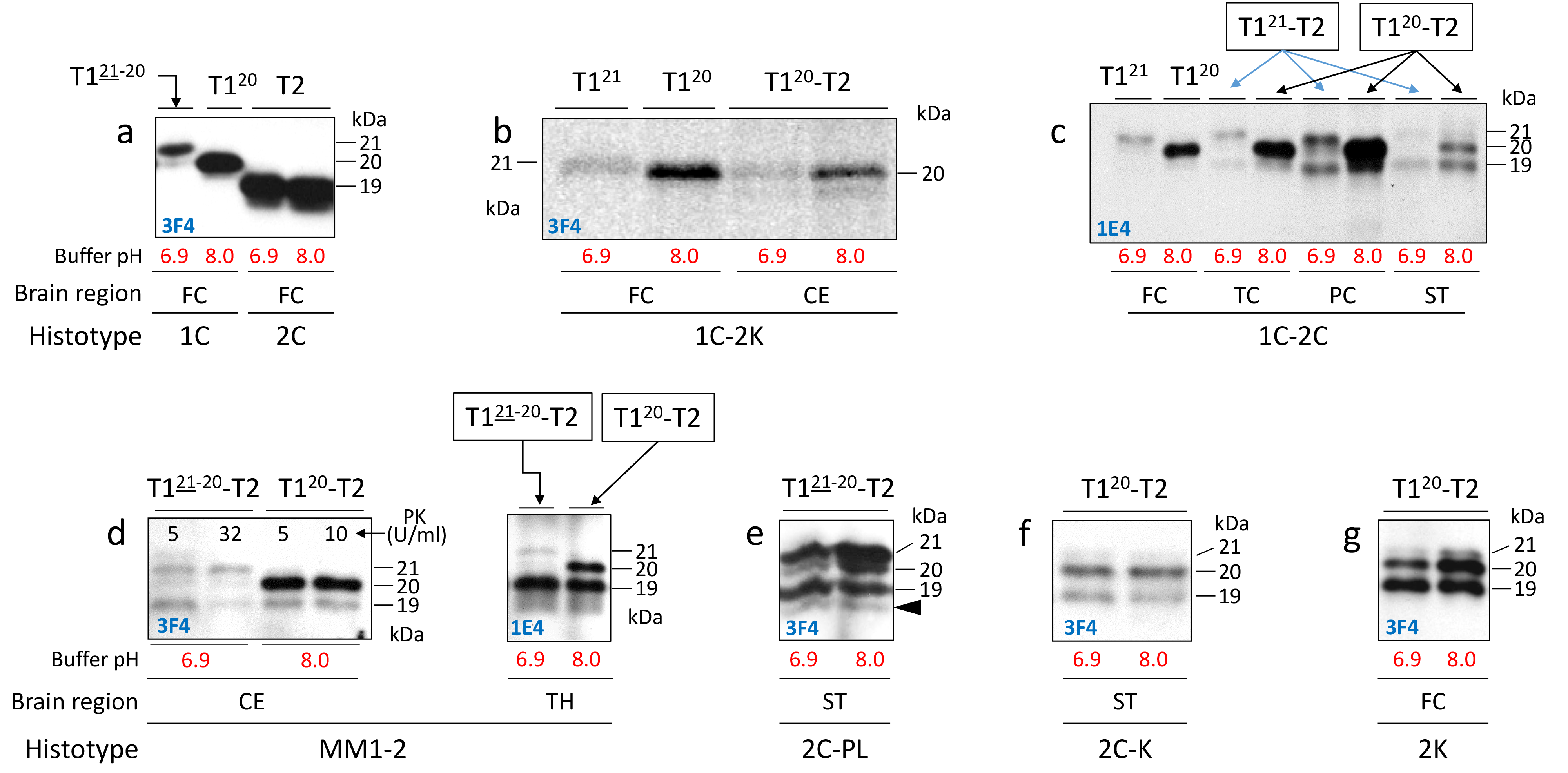

Supplement: Supplementary file 7 — Additional file 7: Fig. S6. Effect of the buffer pH on gel mobility of T1 and T2. Brain homogenates (BH) were prepared with LB100 pH 6.9 and 8.0, digested with PK optima of 32 and 10 U/ml, respectively, and run on a 20 cm-long gel (a, c–g; chemiluminescence) or 8.7-cm long gel (b; near-infrared, LICOR). Only the un-glycosylated isoform of resPrPSc is shown for convenience. a T1 appears as either T121-20 at pH 6.9 or T120 at pH 8.0; T2 is made of a single fragment of ~ 19 kDa at both pHs. b At buffer pH 6.9, T1 migrates to ~ 21 kDa or ~ 20 kDa depending on whether PrPSc is harvested from the frontal cortex (FC) or cerebellum (CE). c resPrPSc migrates as a doublet T121-20 at buffer pH 6.9, or as T120 at buffer pH 8.0. T2 co-exists with T1 in alla brain regions. d resPrPSc appears as T121-20-T2 at buffer pH 6.9, or as T120-T2 at buffer pH 8.0; 5 U/ml PK was used in addition to 32 U/ml (pH 6.9) and 10 U/ml (pH 8.0). e–g Gel mobility of T121-20-T2 (e) and T120-T2 (f and g) is not influenced by the buffer pH; arrowhead, e lower resPrPSc fragment of ~ 18 kDa. FC: frontal cortex; TC: temporal cortex; PC: parietal cortex; ST: striatum; TH: anterior thalamus. T121-20: T121 predominant over T120. [file 40478_2023_1631_MOESM7_ESM.tif]

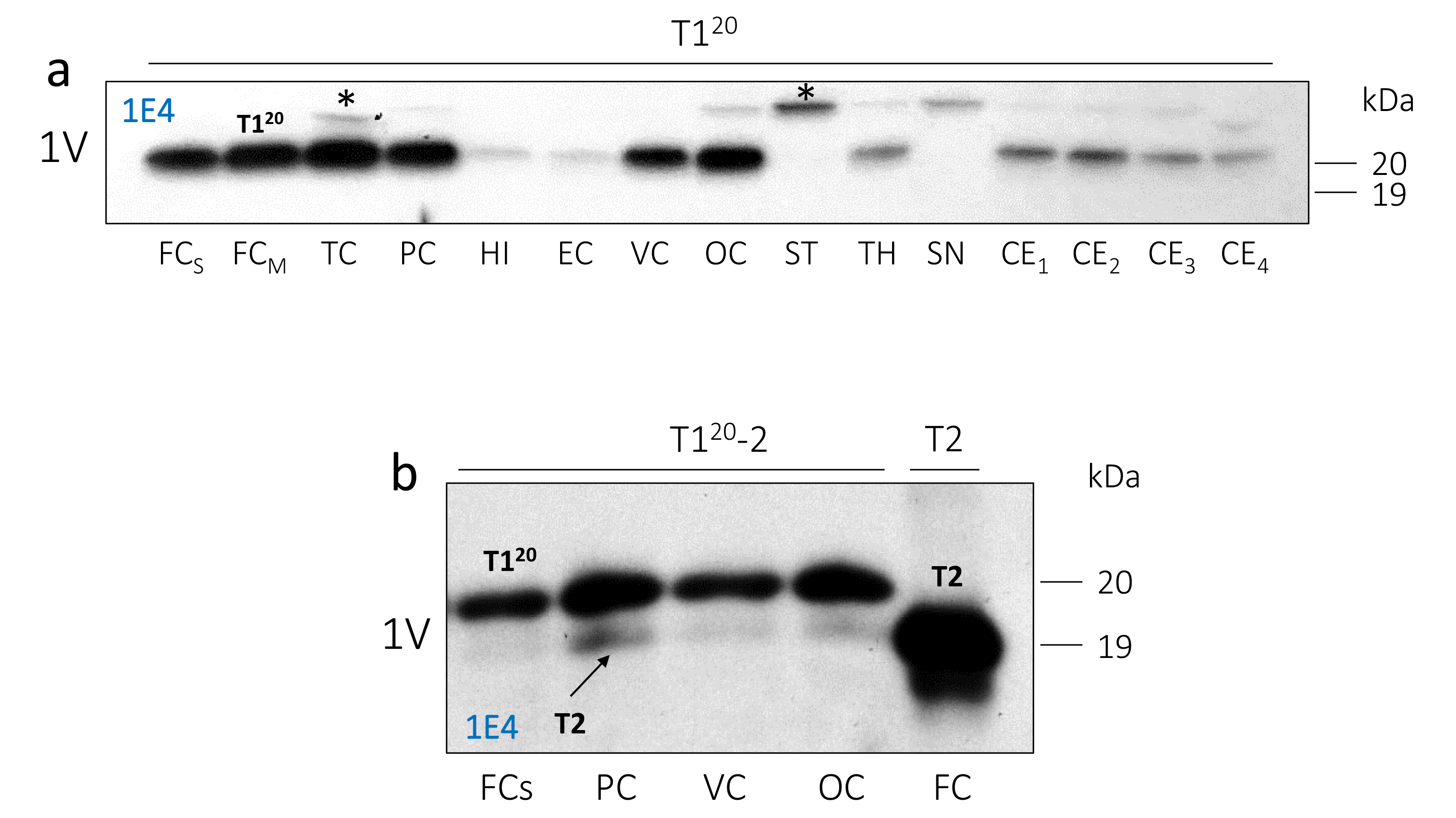

Supplement: Supplementary file 8 — Additional file 8: Fig. S7. Typing of resPrPSc in 1V. Only the un-glycosylated isoform of resPrPSc is shown for convenience. a and b Chemiluminescence (a and b), 8.7- (a) and 20-cm (b) long gels. a Only T120 is detected in a 8.7-cm gel. At longer film exposures, T120 is detected also in the striatum (ST) and substantia nigra (SN) (not shown). b In the cerebral cortex T120 co-exists with a slower migrating band of ~ 19 kDa (arrow) matching the gel mobility of T2. FCs: frontal cortex (cx), superior gyrus; FCM: FC, middle gyrus; TC: temporal cx; PC: parietal cx; HI: hippocampus; EC: entorhinal cx; VC: visual cx; OC: occipital cx; TH: anterior thalamus; CE1-4: four distinct regions of the cerebellum. [file 40478_2023_1631_MOESM8_ESM.tif]

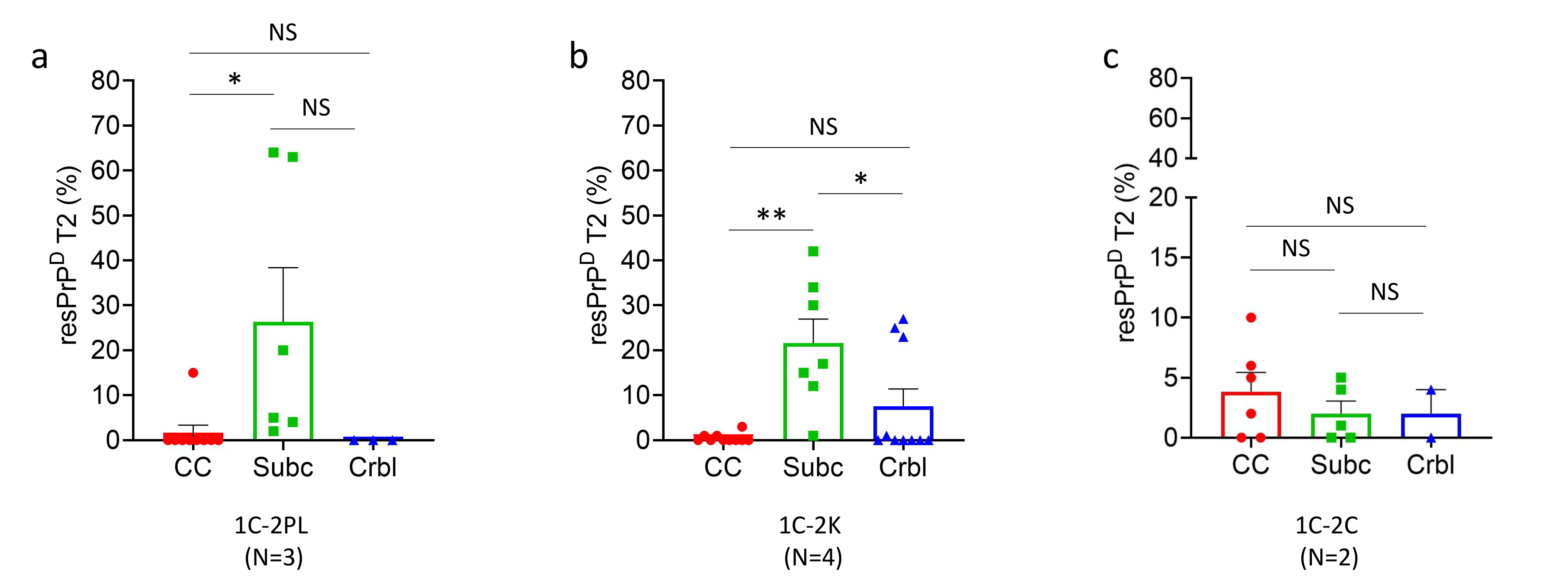

Supplement: Supplementary file 9 — Additional file 9: Fig. S8. Brain distribution of T2 in 1C-2PL, 1C-2K and 1C-2C. a and b In 1C-2PL and 1C-2K, T2 preferentially accumulates in the subcortical regions (Subc) (a and b), and to a lesser extent in the cerebellum (Crbl) in 1C-2K (b). c In 1C-2C, T2 accumulates is similar proportions in each brain compartment; CC: cerebral cortex. *P<0.03-0.05; **P<0.002. [file 40478_2023_1631_MOESM9_ESM.tif]

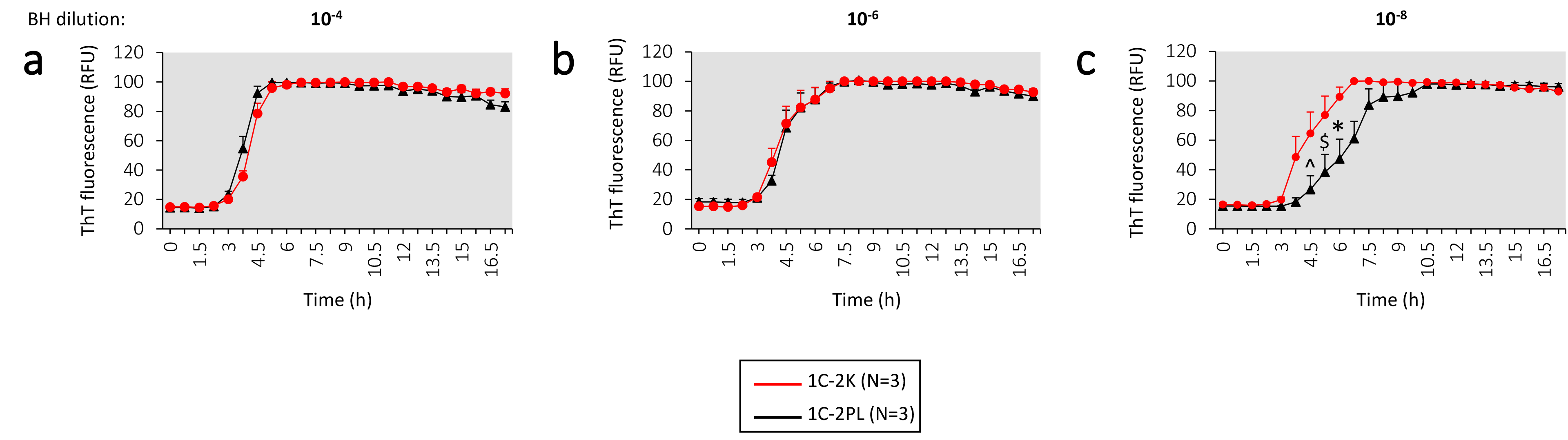

Supplement: Supplementary file 10 — Additional file 10: Fig. S9. RT-QuIC of thalamic PrPSc from 1C-2P and 1C-2K histotypes. a and b Similar seeding kinetics of PrPSc are seen at 10-4 (a) and 10-6 (b) dilutions. c Seeding activity is more efficient at greater dilutions (10-8) in 1C-2K. *P<0.05; ^P = 0.06; $P = 0.08. [file 40478_2023_1631_MOESM10_ESM.tif]
